# On the governing fragmentation mechanism of primary intermetallics by induced cavitation

**DOI:** 10.1016/j.ultsonch.2020.105260

**Published:** 2020-07-24

**Authors:** Abhinav Priyadarshi, Mohammad Khavari, Tungky Subroto, Marcello Conte, Paul Prentice, Koulis Pericleous, Dmitry Eskin, John Durodola, Iakovos Tzanakis

**Affiliations:** a Faculty of Technology, Design and Environment, Oxford Brookes University, Oxford OX33 1HX, United Kingdom; b Brunel Centre for Advance Solidification Technology (BCAST), Brunel University London, Uxbridge UB8 3PH, United Kingdom; c Anton Paar TriTec SA, Vernets 6, 2035 Corcelles, Switzerland; d Cavitation Laboratory, School of Engineering, University of Glasgow, Glasgow G12 8QQ, United Kingdom; e Computational Science and Engineering Group (CSEG), Department of Mathematics, University of Greenwich, London SE10 9LS, United Kingdom; f Tomsk State University, Tomsk 634050, Russia; g Department of Materials, University of Oxford, Oxford OX1 3PH, United Kingdom

**Keywords:** Ultrasonic melt treatment, Cavitation bubbles, Shock waves, Primary intermetallic crystal, Depth sensing indentation, High-speed imaging, Fragmentation

## Abstract

One of the main applications of ultrasonic melt treatment is the grain refinement of aluminium alloys. Among several suggested mechanisms, the fragmentation of primary intermetallics by acoustic cavitation is regarded as very efficient. However, the physical process causing this fragmentation has received little attention and is not yet well understood. In this study, we evaluate the mechanical properties of primary Al_3_Zr intermetallics by nano-indentation experiments and correlate those with in-situ high-speed imaging (of up to 1 Mfps) of their fragmentation process by laser-induced cavitation (single bubble) and by acoustic cavitation (cloud of bubbles) in water. Intermetallic crystals were chemically extracted from an Al-3 wt% Zr alloy matrix. Mechanical properties such as hardness, elastic modulus and fracture toughness of the extracted intermetallics were determined using a geometrically fixed Berkovich nano-diamond and cube corner indenter, under ambient temperature conditions. The studied crystals were then exposed to the two cavitation conditions mentioned. Results demonstrated for the first time that the governing fragmentation mechanism of the studied intermetallics was due to the emitted shock waves from the collapsing bubbles. The fragmentation caused by a single bubble collapse was found to be almost instantaneous. On the other hand, sono-fragmentation studies revealed that the intermetallic crystal initially underwent low cycle fatigue loading, followed by catastrophic brittle failure due to propagating shock waves. The observed fragmentation mechanism was supported by fracture mechanics and pressure measurements using a calibrated fibre optic hydrophone. Results showed that the acoustic pressures produced from shock wave emissions in the case of a single bubble collapse, and responsible for instantaneous fragmentation of the intermetallics, were in the range of 20–40 MPa. Whereas, the shock pressure generated from the acoustic cavitation cloud collapses surged up to 1.6 MPa inducing fatigue stresses within the crystal leading to eventual fragmentation.

## Highlights

• Mechanical properties of extracted intermetallics determined using a nano-indenter.
• High speed imaging captures shock waves breaking intermetallics.
• Shock waves seen to promote crack growth leading to fragmentation.
• In-situ shock wave pressure measurements obtained using a fibre optic hydrophone.
• Subharmonic emissions generate repetitive bending and fracture of intermetallics.

## Graphical abstract

**Figure:**
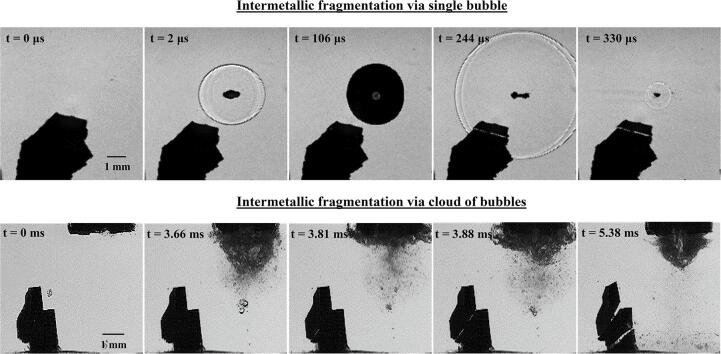

## 1 Introduction

Over the past several decades, aluminium alloys have gained popularity and acceptance as a high strength structural material for applications in the automotive and aerospace industries, also due to their light weight and recyclability. Currently there are sustained efforts to improve material properties of the existing alloys by manipulating their microstructure and minimising the use of expensive and environmentally harmful refinement additives. Recently, processing of liquid alloy melts under the effect of external fields such as melt shearing, electromagnetic stirring and ultrasonic treatment has gained enormous interest owing to effective dynamic microstructural modification upon casting [1]. Among those, ultrasonic cavitation melt treatment (UST) is suggested as an effective method for degassing, filtration, and grain refinement of aluminium alloys [2]. Additionally, UST provides a green, economical and pollution-free alternative approach to a variety of conventional melt processes such as alloying, fluxing etc. [3], [4]. Therefore, over the last few decades there has been considerable efforts in academia and industrial community to control and optimise UST to achieve high efficiency, especially in processing large melt volumes.

The technological and mechanical properties of the as-cast Al alloys can be controlled either by the formation or refinement of primary intermetallic crystals. Under typical casting conditions, the intermetallic crystals grow inadvertently large in shape and size leading to low toughness and ductility of the alloy [5]. Refinement of these primary intermetallics to appropriate sizes (<10 μm) using ultrasonic processing is a promising technique [6]. Using these refined intermetallics as reinforcement particles in typical natural composites such as Al-Si alloys has led to substantial improvement in properties such as hardness, elastic modulus, high temperature stability and corrosion resistance [3], [7]. These primary intermetallics being refined to even smaller sizes (1–5 μm), can act as solidification substrates for Al grains and be very efficient in grain refinement of aluminium alloys even without addition of traditional grain refiners such as AlTiB [8].

Despite the fact that the benefits of UST on various cast alloy systems is well recognised and reproducible [3], [9], [10], basic understanding of in-situ ultrasound fragmentation behaviour of the primary solid phases in the melt is very limited while the governing mechanism of sono-fragmentation is not well understood. Recently, synchrotron X-ray imaging of solidification in real metallic melts under the presence of external fields has attracted attention of researchers and is being widely applied in-situ [11], [12], [13], [14]. Fragmentation of Al_2_Cu intermetallic dendrites in an ultrasonically treated Al-35% Cu alloy was observed by Wang et al. using the in-situ X-ray radiography [12]. Other investigations include the dynamic performance of cavitation bubble in relation to bubble growth mechanism [13]. Interaction of phases formed in Bi-8% Zn alloy with the acoustic cavitation bubbles/flow has also been studied by Wang et al. [11]. However, working with real melts is plagued by difficulties, including the handling of liquid metal melts, recording of multi-phase interactions while restricted by the narrow field of view and a continuously growing solid phase during imaging studies. To overcome these limitations, in-situ observations of organic transparent alloys is the proposed alternative [15], [16], [17].

In-situ grain refinement of organic transparent alloys has been studied using both optical and X-ray imaging techniques [15], [16], [17]. Chow et al. [16] observed that grain nucleation was accelerated under the influence of ultrasonic vibration causing fragmentation of a dendrite by the pulsating and imploding cavitation bubble. In-situ imaging studies on cavitation-induced fragmentation of calcite crystals present in a CaCO_3_ solution revealed that fracture occurred due to the perpetual collapse of the developed bubble cluster [18]. Digital photographic observations of the effect of ultrasonic cavitation on the dendrites formed in a SCN-1- wt% camphor alloy also revealed that the shock waves emitted from imploding bubbles were mainly responsible for the dendritic fragmentation [17]. However, the behaviour of the growing dendrites offers a very limited fragmentation standpoint as their mechanical properties differ significantly from those of solidifying dendrites or intermetallic crystals in real metallic alloys. Moreover, the physical properties of organic transparent alloys are also very different from the liquid metal alloys to a large extent making this analogy inappropriate [11].

Finding a suitable transparent analogue to liquid metals and conducting in-situ imaging experiments at ambient conditions can be very revealing and worthwhile in terms of profound clarity of the interactions. Water, with similar kinematic viscosity and Newtonian behaviour to liquid Al, has been found to have a very similar nature in terms of acoustic cavitation development and bubble dynamics behaviour and is frequently a choice as a transparent analogue while conducting UST studies [2], [19], [20]. Wang et al. [21] recently investigated the cavitation-induced fragmentation and associated fracture mechanism of primary crystals (Si, Al_3_Ti and Al_3_V) formed in Al alloys. This seems to be the most relevant piece of work on the interaction of cavitation bubbles with primary intermetallic crystals to date. Study confirmed that the fracture of the crystals was caused primarily via the fatigue mechanism initiated by the cyclic pressure exerted by pulsating bubbles, possibly triggering a pre-existing crack to grow to a critical size followed by brittle fracture. Yet, this study was performed at moderate frames-per-second levels, and was not supported by measurements of acoustic parameters or mechanical properties. From other studies, it is also anticipated that fragmentation of dendrites in real melts happens by the combined effect of acoustic streaming jet impact [12] and shock waves propagation [22] from imploding bubbles. On the other hand, a recent similar study on the fragmentation of kidney stones suggested that the potential mechanism of stones’ fragmentation is primarily associated with impinged liquid jet followed by powerful shock wave emissions [23].

Implosion of cavitation bubbles and the resulting shock pressure field has been widely studied in water [24], [25], [26], [27], [28], [29], [30]. Cavitation bubbles are generated during the rarefaction phase of the incident ultrasonic wave, grow in size and rapidly collapse when they reach a critical resonance size during the compression cycle producing high-speed jets and local hydrodynamic impact pressures in the range of GPa [31]. However, the exact mechanism leading to the fragmentation of solid phases is still under scrutiny. Understanding the underlying mechanisms of cavitation-induced fragmentation of solid phases will allow us to accurately develop numerical models and advance UST into industrial scale applications [32], [33]. To this end, it is of interest to further investigate the fragmentation behaviour of the intermetallic crystals under controlled cavitation using a single laser-induced bubble event, to study the details of cavitation interaction with the intermetallics and then expand the experiment to continuous ultrasonic processing in order to understand the dynamics of this phenomenon in its full complexity. The acoustic parameters and in-situ observations need to be related to the mechanical properties of the fragmented phases.

In this paper, the mechanical properties of the primary Al_3_Zr intermetallic has been evaluated using depth sensing indentation at room temperature conditions. Subsequently, the fragmentation of primary Al_3_Zr intermetallic under the influence of a single and multiple bubble cavitation has been investigated in deionized water, complemented with in-situ high-speed imaging studies. The fracture mechanism of the intermetallic crystals has been elucidated using the recorded images and validated using critical stress-fracture mechanics. The results reveal for the first time that the fragmentation of intermetallic crystals was essentially prompted by the interaction with shock waves released from imploding bubbles. The fragmentation criterion has also been supported by the pressure field measured by a high-frequency calibrated hydrophone to account for the shock wave-intermetallic interaction.

## 2 Experimental methodology

### 2.1 Extraction of Al_3_Zr and sample preparation

**Fig. 1:**
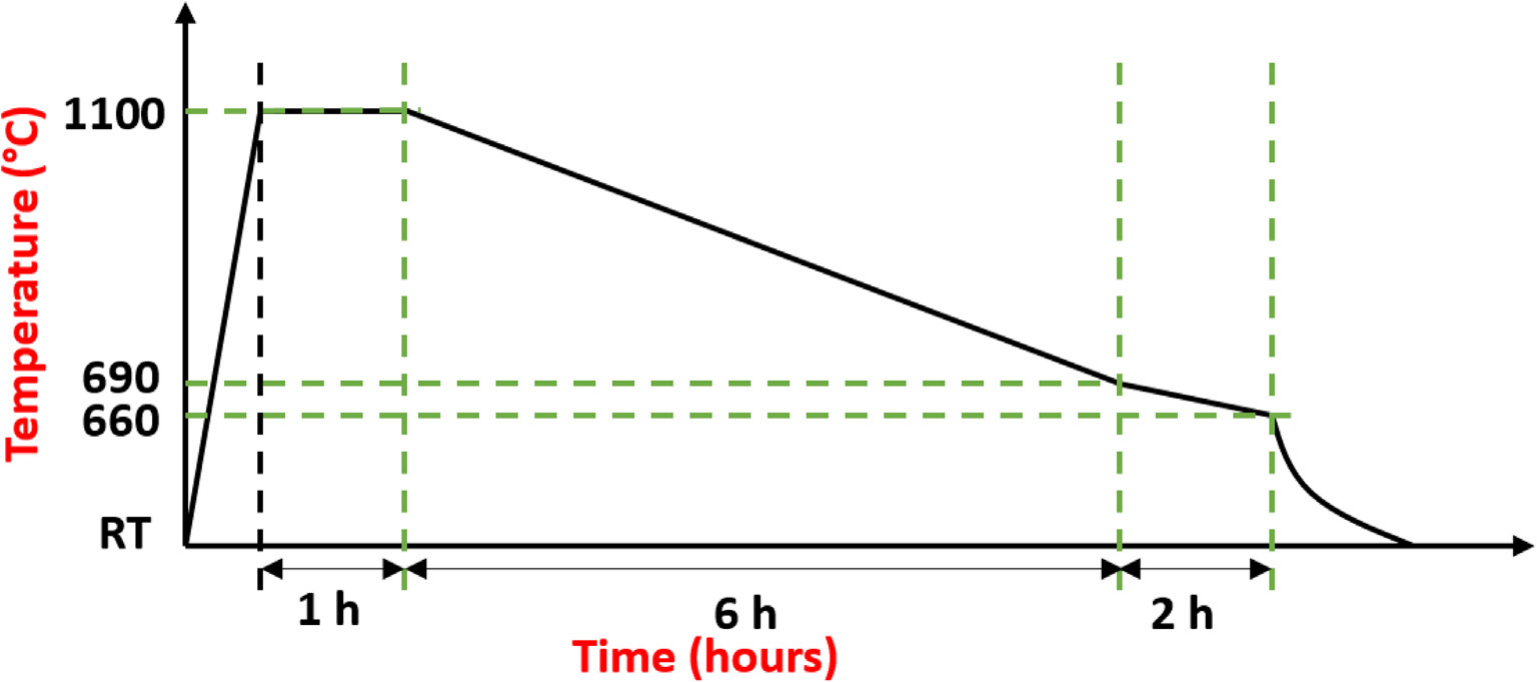
Cooling curve for Al-3 wt% Zr alloy formation.

Around 350 g of an Al-3 wt% Zr alloy was prepared by smelting pure Al (99.97%) and an Al-5 wt% Zr master alloy. The alloy produced was re-melted in an electrical furnace and left to solidify in a graphite mould (Ø=50mm). The alloy melt was cooled to room temperature (RT) based on the schematic thermal cycle shown in Fig. 1, with temperature accuracy of up to 3 K. Cubes of dimension 5 × 5 × 5 mm were cut from the solidified ingot using a silicon carbide rotating blade.

Primary Al_3_Zr intermetallics were then extracted by immersing the cube samples into a 15% sodium hydroxide solution in water for up to 24 hrs. The Al matrix was dissolved into the solution following the chemical reaction:

H2 2Al + 2NaOH + 2H_2_O → 2NaAlO_2_ + 3

**Fig. 2:**
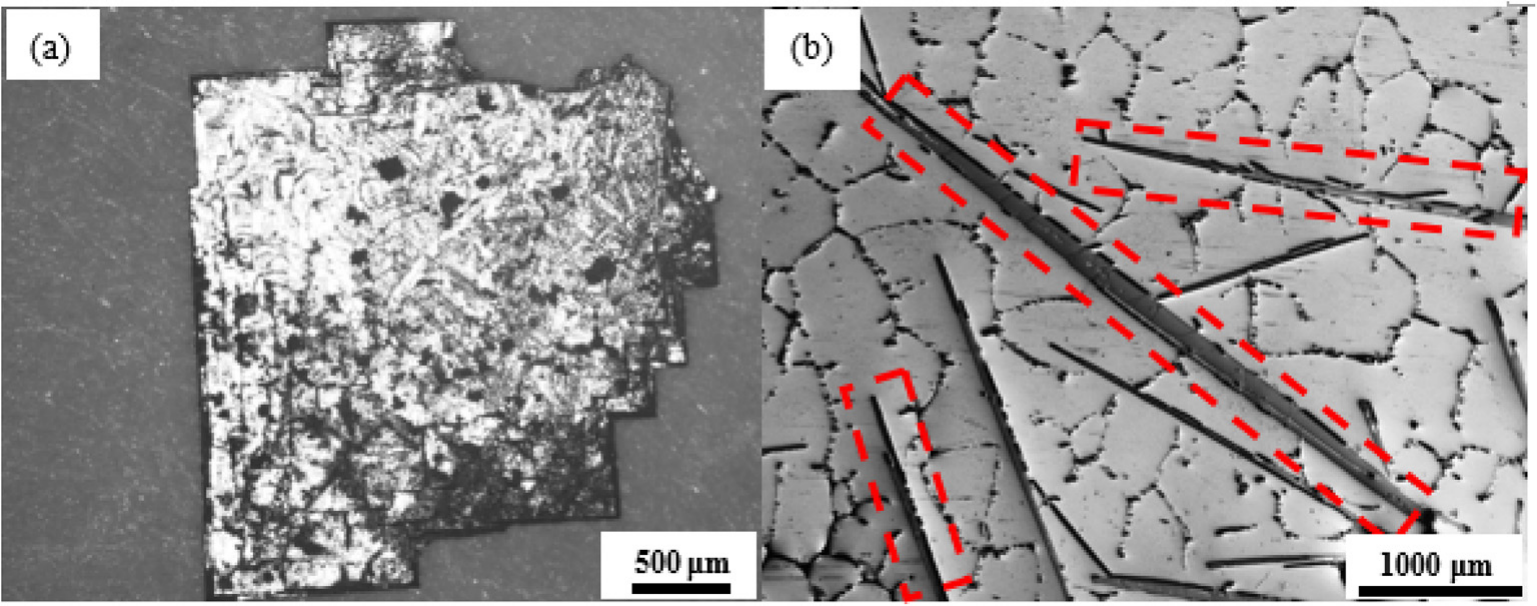
Optical micrographs of Al_3_Zr (a) extracted single crystal, (b) intermetallics embedded in Al matrix.

The solution was then filtered out and the extracted intermetallics were collected and thoroughly rinsed using ethanol and left to naturally dry out for further examination. Morphological image of an extracted Al_3_Zr intermetallic is shown in Fig. 2a.

Part of the ingot was cut longitudinally and sectioned along the central axis from the bottom as the primary intermetallics tends to settle at the bottom of the ingot owing to the slow cooling rate and high density of Al_3_Zr. These sectioned sample were then ground and polished for optical microscopic examination and nano-indentation studies. Fig. 2b shows the morphological images of the Al_3_Zr intermetallics embedded in the alloy matrix (marked in red).

### 2.2 Depth sensing nano-indentation (DSI)

An ultra-high temperature nano-indentation setup (UNHT HTV, Anton Paar Switzerland) working on a principle of active surface referencing was used to analyse the mechanical properties of primary intermetallic Al_3_Zr specimens embedded in Al matrix. This principle employs measuring and referencing axes, each with their own means of computing the applied load and the corresponding displacement. Through independent control of the referencing axis, it is possible to achieve active referencing of the sample to be measured owing to sample-frame deformation and temperature changes. Note that the measurements were conducted at room temperature conditions. The details regarding the instrumentation setup and configuration are provided elsewhere [34]. Successive indentations were performed over the specimen with a continuous loading and unloading speeds of 20 mN/min and a dwell time of 5 sec using a geometrically well-defined Berkovich diamond nano-indenter. The load–displacement characteristics were acquired with a linear loading of 10 mN and an acquisition frequency of 10 Hz. Embedded intermetallics were then subjected for fracture indentation mode using a specifically designed diamond cube corner indenter with a maximum load of 100 mN. At least 5 indents were done for each measurement conditions to achieve statistically reliable data.

### 2.3 Single laser-induced bubble (LIB)

**Fig. 3:**
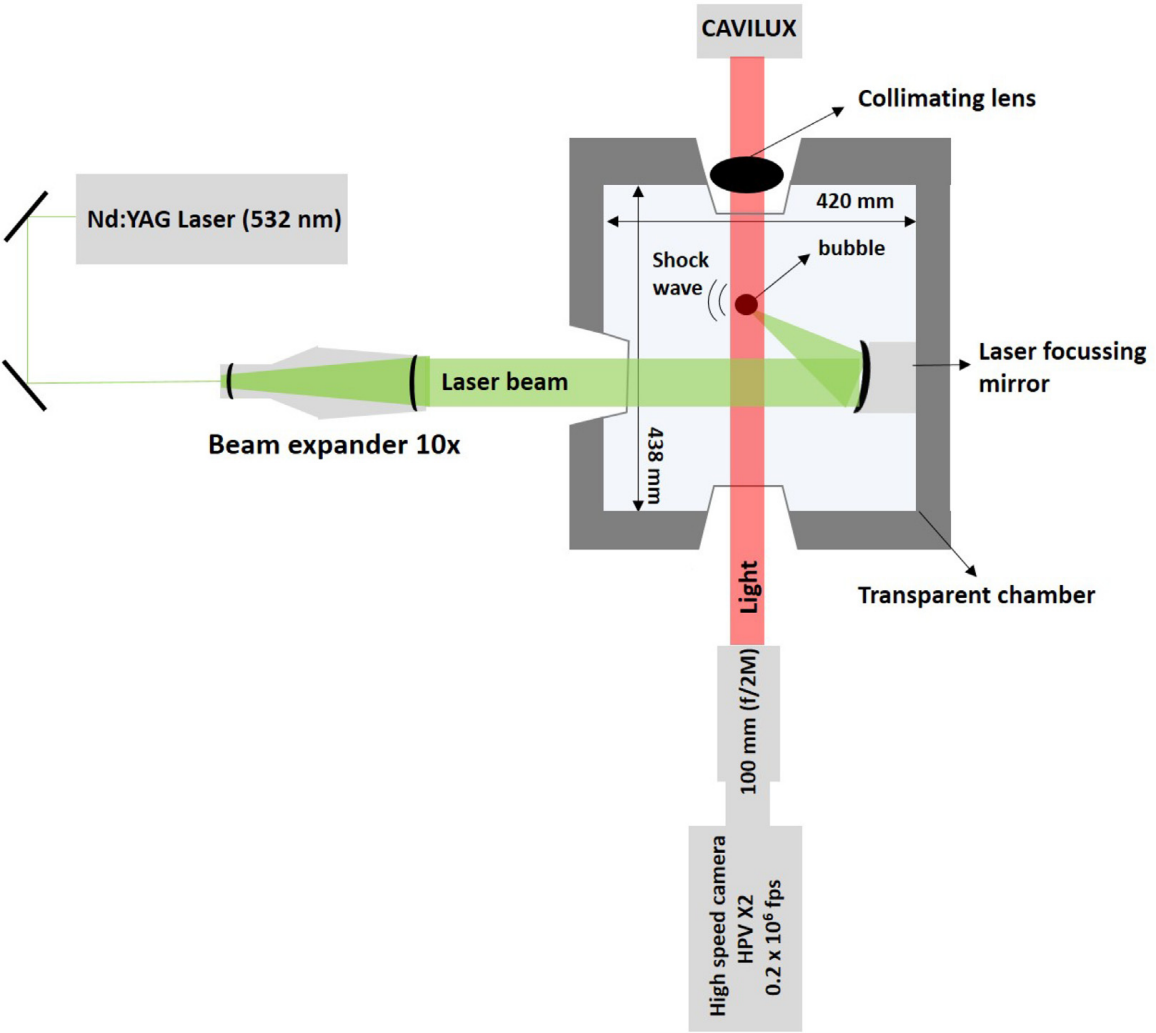
Schematic representation of the experimental setup used for generating laser-induced bubble (LIB).

A Nd:YAG laser system (Nano S 130-10, Litron Lasers, UK) was deployed to generate laser-induced cavitation bubbles by focusing a single high-energy laser pulse of 10.5 ± 1 mJ, with a wavelength of 532 nm (Green beam) and duration of 6–8 ns into distilled water. A transparent custom-built chamber of dimensions 420 mm × 438 mm × 220 mm equipped with a curved mirror to focus the laser beam was used (Fig. 3). The detailed information about the experimental arrangement can be found elsewhere [25]. An extracted intermetallic crystal was then fixed on a steel substrate using a cyanoacrylate adhesive. The attached crystal was placed on a bespoke xyz translational stage and judiciously positioned in such a way that laser breakdown occurs in close proximity to the fixed crystal at a radial distance of 3–4 mm. The standoff parameter *γ* (ratio of the distance of breakdown point from the crystal’s surface and the maximum bubble radius) was deliberately kept in the range of 1–1.5 to ensure that the bubble does not touch the crystal upon expanding. In-situ recordings of the interaction of the single laser bubble with the intermetallic crystal were captured using high-speed shadow-graphic imaging performed at 200,000 frames per second with resolution of 400 × 250 pixels by employing a Hyper vision HPV X2 (Shimadzu, Japan) high-speed camera producing 256 frames for every recorded sequence with shutter time of 200 ns. The illumination and high temporal resolution were provided by synchronous (to frame capture) 10 ns laser pulses of 640 nm (Red beam) via CAVILUX Smart UHS system (Cavitar Ltd., Tampere, Finland). The images (and video in supplementary material) given in this article have been confirmed after 5 similar observations and are found to be repeatable and descriptive.

### 2.4 Ultrasonic processing (USP)

**Fig. 4:**
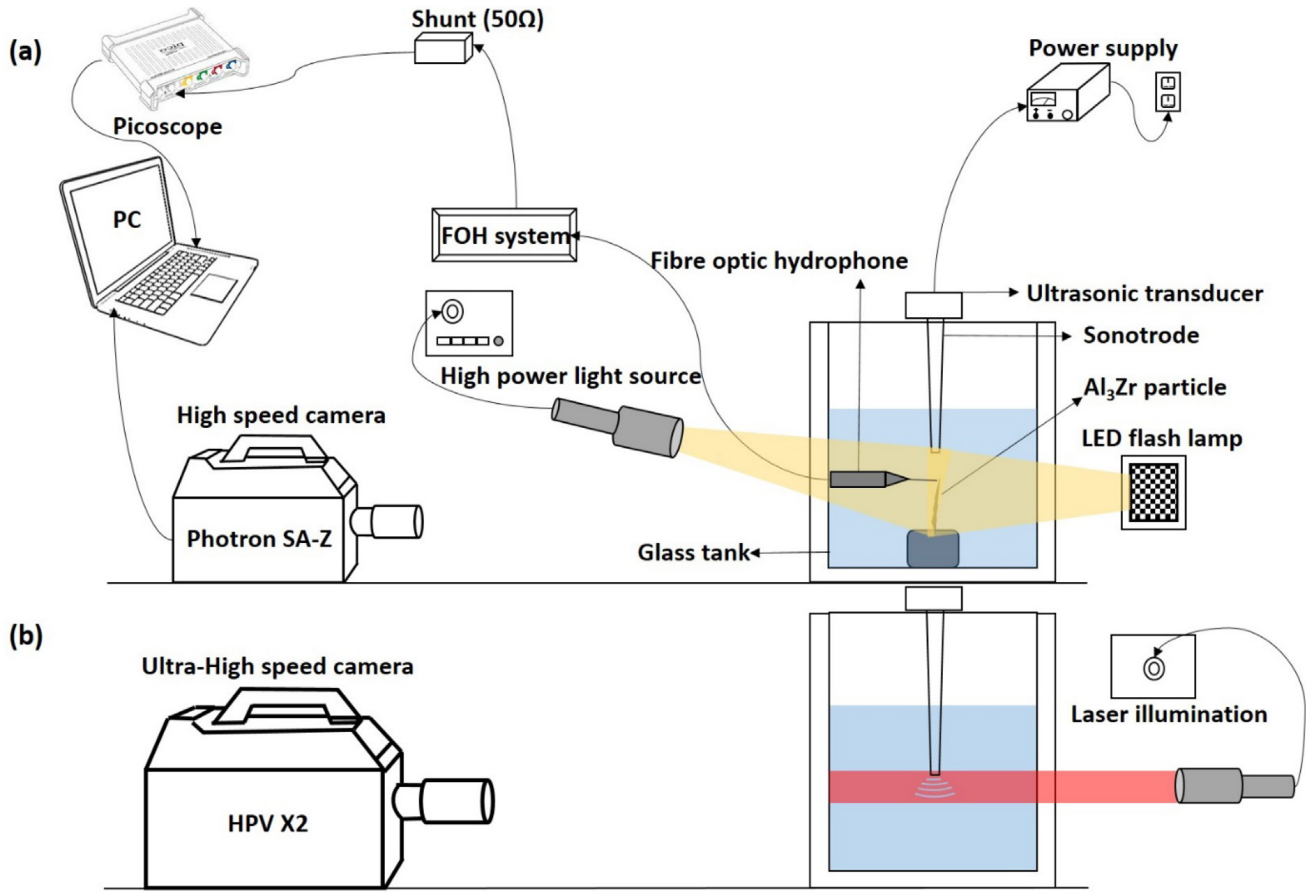
Schematic of the experimental setup involved for (a) sono-fragmentation, and (b) shock wave imaging study.

The extracted Al_3_Zr crystals were fixed onto a steel plate by a cyanoacrylate adhesive and that was deliberately placed on the bottom of the glass tank of dimensions (W × L × H) 75 mm × 75 mm × 100 mm (Fig. 4a). The tank was then filled with de-ionized water up to a height of 75 mm from the base and placed below the 24 kHz ultrasonic horn (Hielscher UP200S Ultrasonic processor) made of titanium with a tip diameter of 3 mm and an adjustable acoustic oscillating amplitude from 42 to 210 μm peak-to-peak at full power (100%). The detailed technical specification of the ultrasonic processor is listed in the manual [35]. The intermetallic crystals were placed precisely 3–4 mm below and at crosswise direction of the sonotrode tip. The sonotrode was immersed approximately 10 mm below the surface of water. The experiments were performed at a chosen peak-to-peak amplitude of 42 μm (corresponding to 20% input power) and conducted under room temperature conditions.

In-situ imaging of interaction between cavitating liquid and primary intermetallic crystals were captured and recorded using high-speed camera (Photron SA-Z). A frame rate of 100,000 fps was chosen for imaging the sono-fragmentation behaviour at a resolution of 640 × 280 pixels with a shutter speed of 8.39 μs. In this case, front light photography was used in contrast to shadow photography in LIB experiments. A combination of a multi LED flash lamp (GS Vitec) and a high-power light source (Karl Storz Power LED 175) from the front and back of the glass tank respectively was utilised in order to achieve maximum illumination in the direction for filming. Fig. 4a shows the schematic of the experimental setup made for sono-fragmentation imaging studies. Minimum of 10 consistent and repeatable observations of the fragmented crystals and their interaction with the cavitating field were recorded in real time.

Fig. 4b exhibits a separate setup that was explicitly used for capturing the propagation of shock waves from the collapsing bubbly clouds using shadow-graphic imaging. The developed cavitation zone nearby the sonotrode surface was recorded at 1 million frames per second in contrast to the crystal fracture observation performed at 100 kfps (due to different time scale of the events) using Hyper Vision HPV X2 (Shimadzu, Japan) ultra-high-speed camera under synchronous 10 ns laser pulses (CAVILUX) illumination in order to capture and quantify the highly dynamic behaviour and propagation of shock waves. Multiple sets of shock wave fronts for a period of 0.25 ms were captured, analysed and related to the fragmentation mechanism (typically occurs after 8–10 ms).

### 2.5 Acoustic pressure measurements

**Fig. 5:**
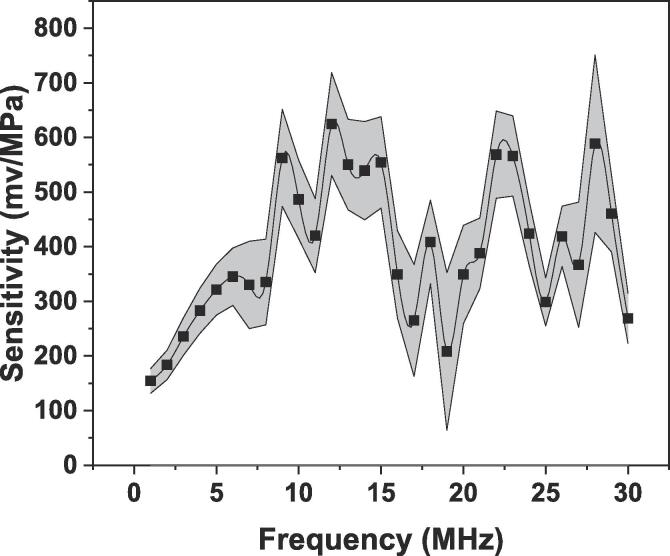
Sensitivity of FOH calibrated in discrete steps of 1 MHz between 1 and 30 MHz bandwidth frequencies, with calibration uncertainty represented as grey shading.

A fibre optic calibrated hydrophone (FOH) device, with a 125 μm probe sensor (Precision Acoustics Ltd., Dorchester, UK) was integrated to the experimental setup as shown in Fig. 4 to monitor the pressures at MHz frequencies associated with transient cavitation and shock wave emissions. The sensor was connected to the digital oscilloscope-3204D (Pico Technology) through the 50 Ω output of FOH system to capture the acoustic signal generated from the ultrasound device. The electronic connection of the sensor is schematically represented in Fig. 4a. The hydrophone was calibrated between 1 and 30 MHz at increment of 1 MHz using a PVDF (Polyvinylidene fluoride) membrane hydrophone (0.4 mm diameter) with a sensitivity and its associated uncertainties (grey region) varying as shown in Fig. 5. The shock wave pressure was recorded at locations close to the tip (±0.2 mm) of Al_3_Zr crystal placed at 3 mm vertically below the sonotrode (under) along the symmetry axis and 4 mm in the transverse axis (side).

The real time acoustic signal monitoring was captured using the Picoscope data acquisition software. The raw hydrophone voltage signal was obtained over the period of 2 ms (~48 acoustic cycles) with a sampling rate of 500 MS/s (Mega samples per second). The raw data was acquired after the steady state condition had been attained. The Fast Fourier Transform (FFT) of the voltage–time signal that was applied to obtain the pressure signal followed the deconvolution process as previously described in our work [36] and in [37]. The pressure values were averaged over 60 waveform signals of 2 ms each to ensure repeatability of the results while noise was subtracted from the raw signal.

## 3 Results and discussion

### 3.1 Nano-indentation experiments

Formation and growth of the primary intermetallic phases to a certain size in the solidifying melt predominantly affects the material integrity and mechanical properties of the as-cast alloy. The ultrasound-induced fragmentation potential and refinement of these primary crystals depends, on one hand, on the operating conditions of UST, and, on the other hand, on the fracture strength of the crystal. Knowing the latter is a vital input in deciding the acoustic power requirements for efficient and controllable ultrasonic fragmentation. Yet these properties are largely unknown. To fill this gap, we performed this part of the study.

**Fig. 6:**
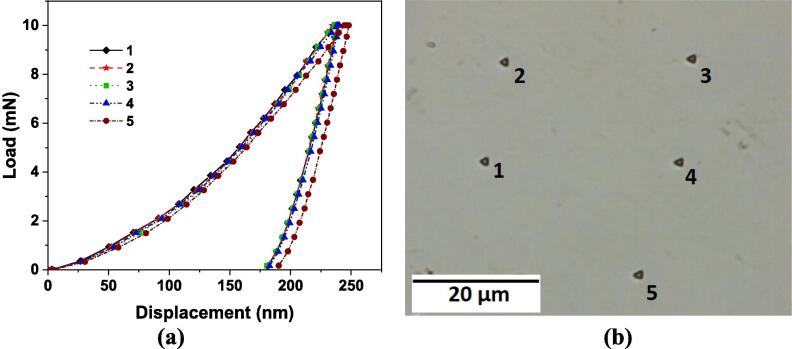
(a) P-h curve with maximum and final penetration depth of 240.8 ± 4.14 and 183.1 ± 4.06 respectively, and (b) corresponding indentation marks on Al_3_Zr crystal with load of 10 mN.

Mechanical properties such as elastic modulus, nanohardness and fracture toughness of the primary Al_3_Zr intermetallic have been evaluated here experimentally and compared with the values in literature obtained from first principle calculations [38]. Fig. 6 shows 5 load–displacement (*P*-*h*) profiles of the Al_3_Zr intermetallic and their corresponding indentation marks obtained for a maximum load of 10 mN at room temperature using the nano-indentation setup of section 2.2. The mechanical properties have been evaluated from the P-h curve using the method introduced by Oliver and Pharr (O&P) [39], [40]. To obtain fairly accurate values of elastic modulus and hardness from this P-h curve, it is important to measure experimentally certain parameters such as the maximum displacement (*h_max_*), permanent/final depth of penetration (*h_f_*) and the contact stiffness of unloading curve (S=dP/dh). The indentation depth in Fig. 6a shows substantial elastic recovery (~24%) equivalent to that of quartz [41] with a residual penetration depth of almost 183 nm, which strongly points towards the elastic/plastic behaviour of the crystal being indicative of a typical hard and brittle material.

Using the model suggested by O&P, the hardness (*H*) and elastic modulus (*E*) were evaluated and found to be in the range of 7.2–7.6 GPa and 194–206 GPa respectively. The results on material properties of Al_3_Zr based on our experimental measurements are given in Table 1 along with some reference data. The values are in good agreement with the current literature data estimated from the first-principle method [38], [42], [43], [44], [45], [46]. Fracture toughness (*K_c_*) of the intermetallic was also determined from the following equation given by the Lawn Evans Marshall (LEM) model [47], [48]:

(1) $$K_{c}=\alpha \times {\left(\frac{E}{H}\right)}^{0.5}\times \left(\frac{P}{c^{3/2}}\right)$$

where, *P* is applied indentation load, *c* represents the contact size, *α* is the constant dependent on indenter geometry, material’s Poisson’s ratio and *E/H* ratio was taken as 0.036 for a cube-corner indenter. *K_c_* of the Al_3_Zr intermetallic was experimentally estimated to be nearly 1 MPa√m agreeing well with the value found in literature [49], see Table 1.

**Table 1:** Measured mechanical properties of Al_3_Zr crystal using DSI technique.

**Mechanical Properties**	**This work**	**Std. dev.**	**Other work**
Hardness (GPa)	7.4	0.2	6 [45]
Elastic Modulus (GPa)	200	5.8	197.6 [38] 201.8 [42] 193.0 [44]
Fracture Toughness (MPa√m)	1.1	0.1	0.85 [49]

These values have been utilised later for accurate determination of fracture mechanics.

### 3.2 Laser-induced bubble (LIB) experiments

#### 3.2.1 Observations of fragmentation

**Fig. 7:**
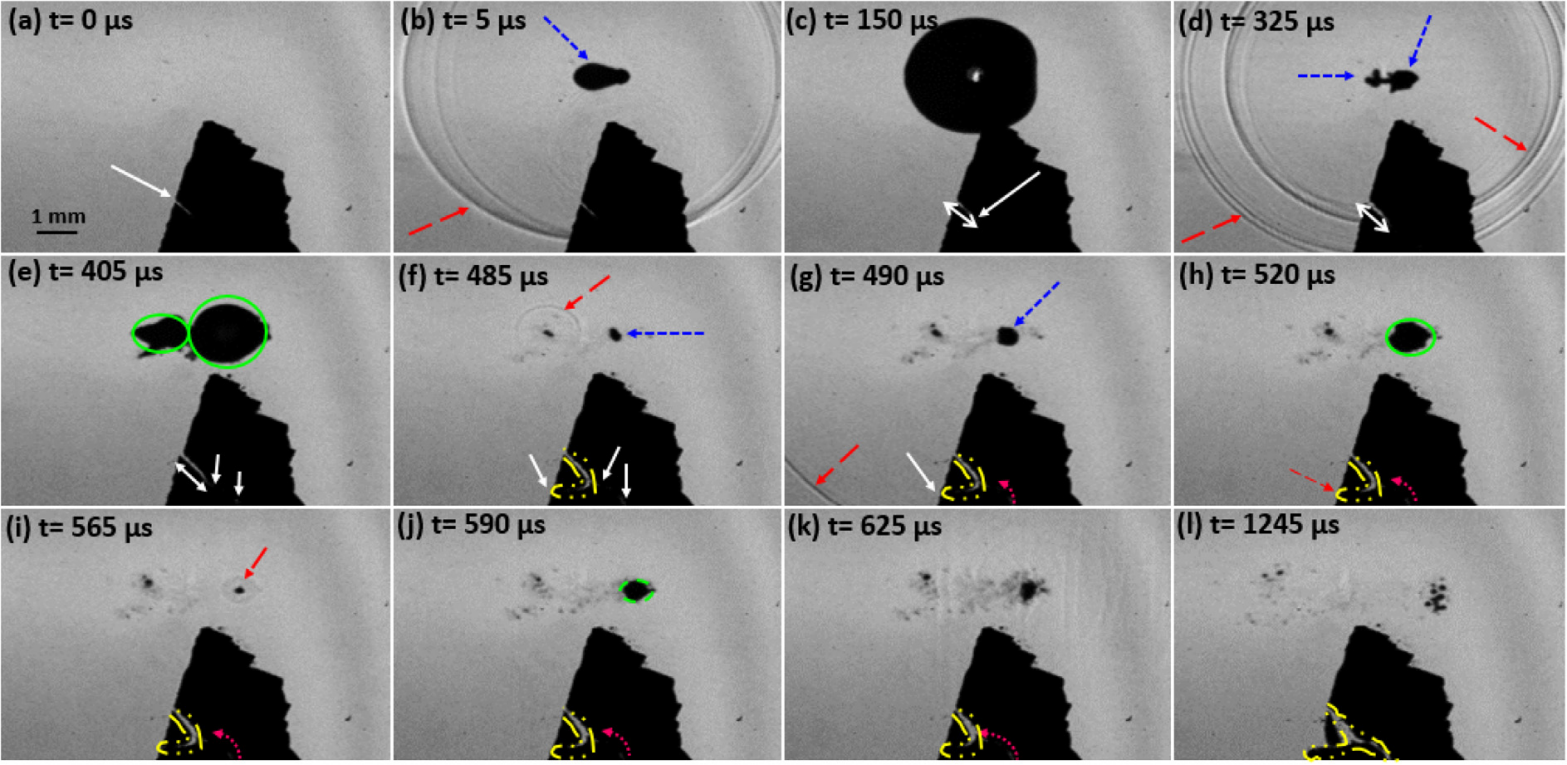
High-speed imaging sequence showing complete fragmentation of primary Al_3_Zr intermetallic particle (pre-existing crack) upon interaction with a shock wave from a laser nucleated single bubble recorded at 200 kfps. The supplementary video can be accessed with the online version of the article as video 1.

A sequence of images captured using high-speed filming of primary Al_3_Zr intermetallic fragmentation is shown in Fig. 7(a-l). An associated video corresponding to this sequence of images (File name: video 1) is accessible with the online version of the article. These real time images demonstrate the interaction of a single laser-induced cavitation bubble with an Al_3_Zr intermetallic crystal leading to its fracture. Fig. 7a shows the studied intermetallic crystal of approximate dimension 3.1 mm × 3.5 mm × 0.06 mm (L × H × W) with a pre-existing crack length of 0.5 mm (indicated with a white arrow) at time t=0 μs. After about 5 μs, an optical breakdown is triggered using the laser energy source causing bubble nucleation (marked with blue arrow) and release of continuous fronts of shock waves propagating with both compressional and rarefaction phases (indicated with red arrow) within the liquid as shown in Fig. 7b. The laser-induced bubble then undergoes the growth phase from t=5 μs to t=150 μs, expanding up to a radius of 1.5 mm as illustrated in Fig. 7 (b to c) in response to the energy disposition of the liquid medium. The crystal interaction with the first set of generated shock wave fronts causes the pre-existing crack (at a distance of approximately 3.3 mm from the bubble centre) to propagate through the intermetallic thereby increasing its length (indicated with white double arrow). Thereafter, the inertia of the host medium causes the bubble to shrink and collapse asymmetrically at t=325 μs, following the release of next set of shock wave fronts scattering within the liquid.

Following the first collapse, a smaller (secondary) bubble is generated by partially splitting from the main (mother) bubble (encircled in green) with sufficient energy to follow its growth and collapse cycle till a complete separation occurs. The secondary bubble then expands to its maximum size until t=405 μs causing the crack to further propagate and a secondary crack to develop (indicated with a white arrow) at the bottom of the intermetallic crystal as shown in Fig. 7e. The smaller bubble then completely splits up from the primary bubble followed by a rebound growth and shrinkage phase and subsequent release of shock waves (indicated with red arrow) upon its final collapse as shown in Fig. 7f. To this effect, the crack rapidly propagates to the other end causing complete fracture of the crystal from the bottom left side (indicated with a yellow boundary). Just after next 5 μs, the primary bubble collapses to generate another set of shock wave fronts (indicated with a red arrow) propagating through the liquid as shown in Fig. 7g. Consequently, the secondary crack can be seen spreading from the bottom part to merge with the primary one (marked with red curved arrow) as shown in Fig. 7(h-l). Multiple rebounds and collapses of the primary cavitation bubble happen until the bubble’s energy is entirely dissipated in the medium and it finally disintegrates into tiny clusters of bubbles as shown in Fig. 7l. Even though it seems from the sequence of images in Fig. 7 that crack propagation in the crystal results from an immediate bubble collapse event, this may not be entirely true owing to a hysteresis induced from the signal delay after the laser is essentially triggered. Therefore, what may appear as the consequence of crystal-shock wave interaction from a rebound collapse process, could actually be a result of preceding shock waves generated through previous cavity collapses i.e. the damaging effect of the propagating shock fronts on the intermetallic produced after the 2nd and 3rd rebound collapses at t=485 μs and t=565 μs, respectively, in reality might have induced either from the prior released shock waves t=5 μs and t=325 μs or through their synergistic response. It is also likely that the shock fronts generated from the rebound collapses are too weak to cause any severe damage to the crystal compared to the initial high energy disseminated shocks. It has been observed that a split cavitation bubble (as in the present case) produces multiple shock waves with separate centres in the subsequent breakdowns [50]. So, it is fair to say that the whole crystal fragmentation is essentially a reaction to cumulative shock waves (~5 shock fronts typically monitored after the final collapse) impact from a single laser bubble. Similar response was observed in all other cases of single laser bubble experiments where fragmentation essentially occurred from the split cavitation and asymmetric rebound producing multiple shock waves caused by the residual asymmetry during implosion as achieving a perfect spherical shape of the bubble is barely possible [51]. It is also evident from Fig. 7 that since there was no direct physical interaction of the laser-induced bubble with the intermetallic crystal with standoff parameter *γ* being equal or greater than 1 (see supplementary video 1 for better clarity), the intermetallic fragmentation was solely a result of repeated shock wave cyclic fronts emitted from the breakdown (nucleation) and successive collapse phases of the laser-induced bubble. Also, it is evident from the high-speed images that the crystal underwent almost instantaneous fragmentation befalling in less than a millisecond time. It is worth mentioning here that the asymmetrical collapse of the bubble generated multiple shocks that interacted with the fixed sample leading to almost instantaneous brittle fracture. This type of collapse replicates the real shock pressure conditions occurring in continuous sonicated environments as shown later in section 3.3.

#### 3.2.2 Analysis of fracture mechanics of the studied crystals

An attempt to quantify the observed fragmentation mechanism by means of basic fracture mechanics was made. The fragmentation mechanics has been applied in terms of critical stress required to initiate pre-existing crack propagation that completely fractures the intermetallic crystal upon interaction with the laser-induced bubble.

As previously mentioned in section 3.2.1 the predominant fragmentation mechanism is the surge of shock wave pressures upon bubble collapse. Therefore, it is essential to estimate the strength of shock waves in relation to their pressure amplitude. The following equation suggested by Vogel et al. [24] provides the variation of the pressure amplitude (*P_r_*) of the emitted shock wave from a spherical bubble collapse against the propagating distance, *r*.

(2) $$P_{r}=c_{1}\rho_{o}u_{s}\left(10^{\left(u_{s}-c_{o}\right)/c_{2}}-1\right)+P_{\infty }$$

where *ρ_o_* represents the density of the surrounding medium (water) before the emission, *u_s_* denotes the shock wave velocity measured from the *r(t)* curve, *c_o_* corresponds to the speed of sound in water, *c_1_* and *c_2_* are empirical constants equal to 5190 m/s and 25306 m/s respectively as defined in [52], and *P_∞_* indicates the hydrostatic pressure.

**Fig. 8:**
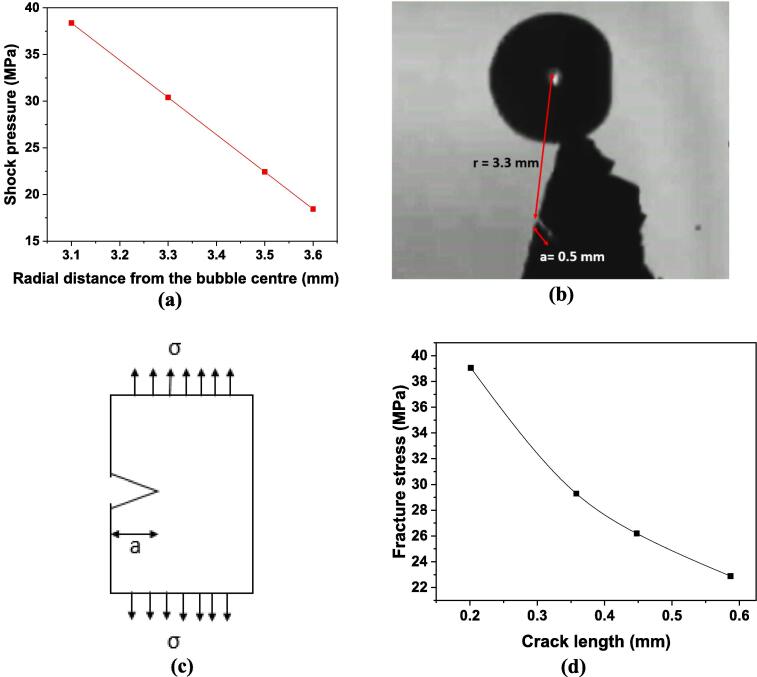
(a) Calculated shock pressure magnitude, (b) image of Al_3_Zr crystal showing crack length and its distance from the bubble centre, (c) schematic representation of stresses acting on single edge notched plate, and (d) theoretical fracture stress required for its fragmentation.

The maximum shock wave velocity (*u_s_*) for the optical breakdown through laser energy of 10 mJ and 6 ns pulse was reported to be almost 3 times the speed of sound in water [24]. Considering the velocity to be approximately 4500 m/s, the shock pressure amplitude was determined using Eq. (2) to be around 7000 MPa. Additionally, using a far-field hydrophone, the shock pressure amplitude, by a similar in nature and energy capacity bubble (10 mJ), was measured in the range of 2.5–10 MPa at a radial distance of 4–4.5 mm from the bubble centre [24], [26]. It has been previously reported that the pressure amplitude close to 100 MPa decays approximately as *r^-1^* nearby the shock front [24], [53]. On a logarithmic scale, the pressure amplitude initially attenuates in an inverse parabolic fashion (*r^-2^*) up to a distance of 300 μm with a value close to 150 MPa and then reduces linearly with the propagating distance until the shock front completely dissipates in the medium [24]. Using these empirical relationships, the shock pressure at a distance of 300 μm from the laser bubble centre was linearly interpolated to a radial distance of 3.3 mm (the distance of the pre-existing crack from the emission centre) and was estimated to be about 31 MPa. The shock pressure amplitude was estimated to vary between 20 and 40 MPa for other specific positions of the crystal placed away from the bubble centre (Fig. 8a) through careful interpolation, keeping in mind the linear trend of the pressure attenuation.

It is to be noted that these predicted pressure values are for an ideal case of a symmetric bubble collapse that produces a single shock wave. It has been reported that the pressure amplitude of the propagating shock waves emitted from an asymmetric bubble collapse at a distance of 3 mm from the plasma centre is in the range of 4–11 MPa depending upon the focussing angle of the laser beam [51]. This variation is also indicative of the fact that the emitted shock wave is formed through superimposition of signals originating from diverse locations across the elongated plasma region. Therefore, the shock pressure obtained for a spherical bubble collapse clearly exceeds the pressure signal generated from an asymmetric collapse.

To relate the induced shock pressures to those required for the complete fracture of the crystal, the critical fracture stress was determined based on the typical Griffith criterion for a single edge notched Al_3_Zr intermetallic (Fig. 8b) assuming that Mode I fracture (Fig. 8c) is applicable [54]:

(3) $$K_{c}=C\sigma \sqrt{\pi a}$$

Here, *K_c_* represents the fracture toughness of the intermetallic crystal, *a* corresponds to the crack length of the pre-existing notch, *C* is a constant parameter depending on the crack length, and *σ* denotes the stress needed for complete fracture of the intermetallic. For the present case, the value of *K_c_* was considered to be 1.1 as obtained from Table 1, the crack length, *a* is taken to be 0.5 mm based on the direct observation, and the constant *C* is taken as 1.12 [55]. Using Eq. (3), the critical stress that would result in the brittle fracture of Al_3_Zr crystal with a known crack length was estimated to be around 25 MPa. The required fracture stress for crystals having crack length between 0.2 mm and 0.6 mm was in the range of 22–40 MPa (Fig. 8d). Therefore, in the ideal case of a spherical collapse, the generated shock pressure is sufficient to break the crystal; however, in our case where multiple collapses are observed due to collapsing asymmetry (elongated plasma breakdown) [52], [56], the distributed shock pressure is expected to be comparatively less. Moreover, this exaggeration of the maximum pressure generated from a single shock wave compared to multiple pressure shocks is also in good agreement with experimental measurements as discussed elsewhere [26], [51].

Nonetheless, due to the observed almost instantaneous breakage it seems that the pressures generated by shock waves are sufficient to overcome the critical fracture stress of up to 40 MPa as estimated by Griffith’s criterion. Also, it is possible that the required stress for fragmentation could be even less than estimated, as the pre-existing crack (as shown in Fig. 7a) branches into two new cracks (Fig. 7l) thereby failing not just as per Mode I but by a combination of other fracture modes that the crystal experiences. This implies the presence of other pre-existing flaws facilitating the crack propagation with a multi-directional tendency. Therefore, the resulting multiple shock wave fronts emitted from the collapse of a laser-induced bubble are sufficient to produce brittle failure of an Al_3_Zr crystal on the spot.

### 3.3 Ultrasonic cavitation experiments

Having identified the governing fragmentation mechanism of intermetallics exposed to cavitation impacts, next step is to investigate the fracture of primary Al_3_Zr crystals (with and without a noticeable pre-existing crack) when subjected to ultrasonic cavitation induced by a sonotrode.

#### 3.3.1 Observations of fragmentation

**Fig. 9:**
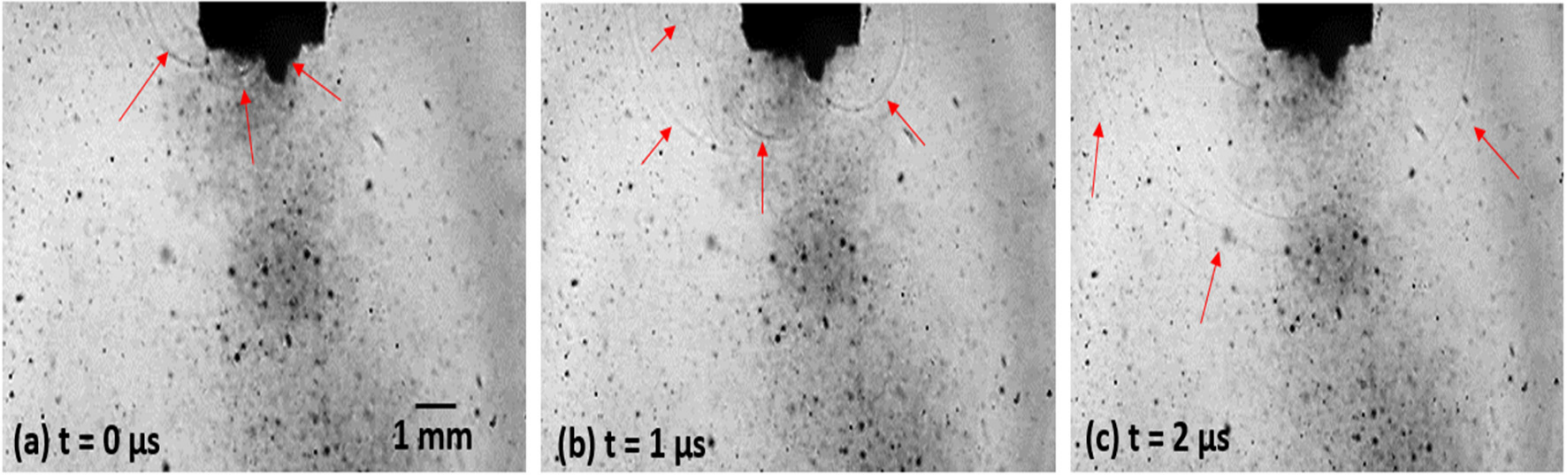
Images of ultrasonic cavitation and shock wave propagation generated from a 3 mm sonotrode recorded at 1Mfps. The supplementary video can be accessed with the online version of the article as video 2.

To start with, the sources of shock waves emissions in this dynamic and highly transient sonicated environment were monitored. Fig. 9 shows typical series of images of an already developed cavitation zone at the tip of the sonotrode captured at 1 Mfps using the integrated laser illumination. It is clear that the cavitation phenomena occurring below the sonotrode emit shock waves in a consistent pattern. The red arrows show the emitted shock wave propagating through the liquid at definite time intervals. An associated video corresponding to this sequence of images (File name: video 2) is accessible with the online version of the article. We observe that the tip of the sonotrode is covered by a cavitation cloud which undergoes repetitive expansion and collapse oscillations thus emitting shock waves. The majority of these shocks are generated from the edges: at most locations of the sonotrode surface there is interference of the acoustic waves from adjacent point sources and, therefore, it is only at the edges where there are uncancelled horizontal components and no shielding from the sonotrode [57], resulting in diffractive field effects and spherical spreading of the shock waves. It has been noticed that the collapse of inertial cavitation cloud against the sonotrode face and interaction of the pressure fields within this cloud will also generate complex pressure waves propagating outwards from the perimeter of the sonotrode tip [58]. video 2 footage shows that around 4 shots of multiple shock wave fronts are released on average in every 0.25 ms from different locations in the vicinity of the ultrasonic source upon collapse of bubbly clouds. It has been argued that these released shock waves have the potential to damage/break the solid crystals in the surrounding area [59], [60], [61]. However, the lack of solid evidence in literature have imposed restrictions for the establishment of such a hypothesis.

**Fig. 10:**
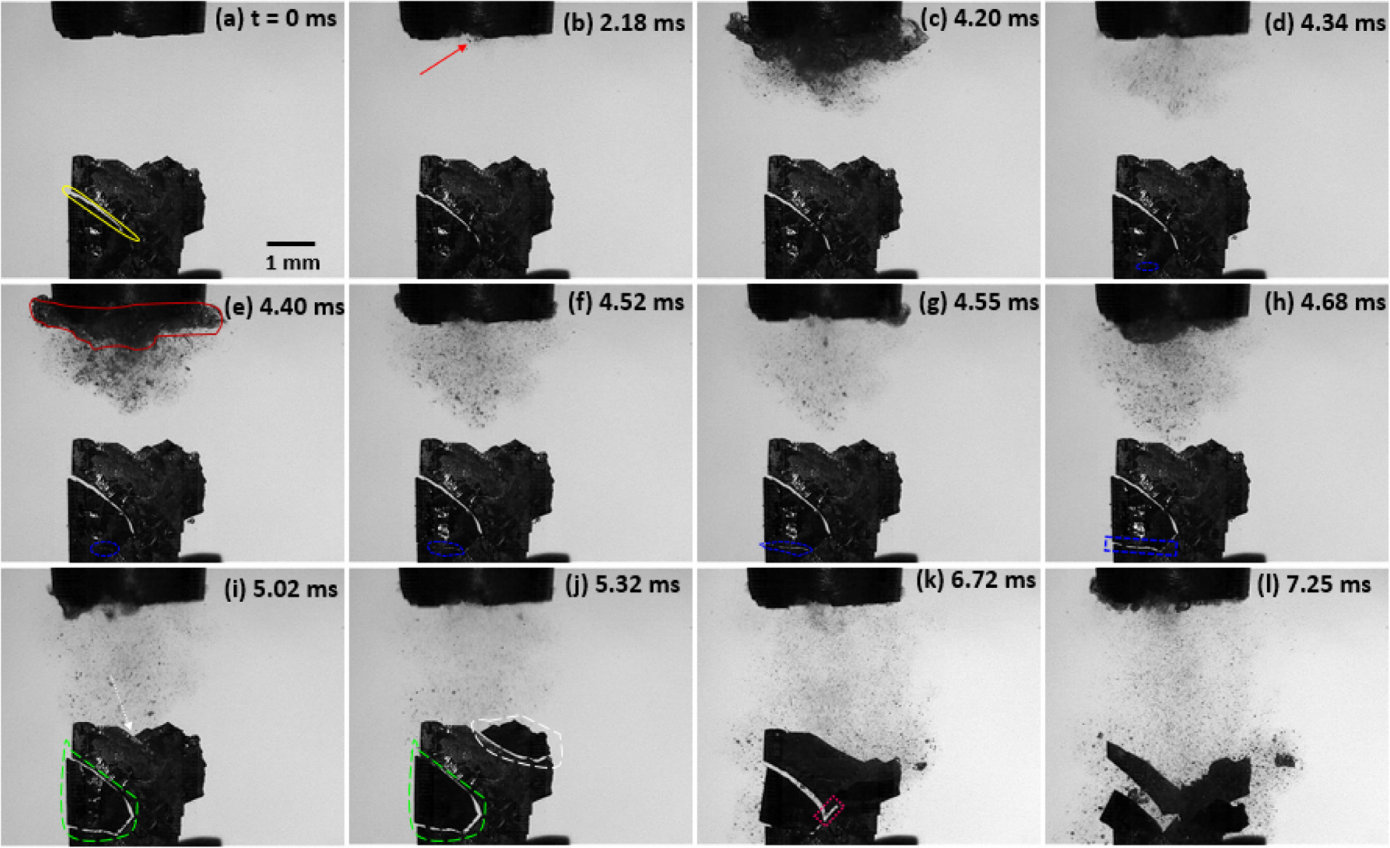
High-speed images of fragmentation of primary Al_3_Zr (single edged notch) crystals captured at 100 kfps when subjected to ultrasonic cavitation. The supplementary video can be accessed with the online version of the article as video 3.

Fig. 10 shows the frame by frame fragmentation process of a single edge notched Al_3_Zr crystal by means of acoustic cavitation. An associated video corresponding to this sequence of images (File name: video 3) is accessible with the online version of the article. In this case a lower frame rate speed was deployed using light illumination as explained in section 2.4. The first frame (t=0 ms) exhibits a well-illuminated typical Al_3_Zr crystal of dimension approximately 2.6 mm × 2.8 mm × 0.06 mm (L × H × W) having a crack/notch length of about 1.8 mm (encircled in yellow). Prior to switching on the ultrasound, it has been made sure that there is no presence of microbubbles (greater than10 μm, camera lens cannot resolve smaller size bubbles) in the vicinity of the crystal. Fig. 10b shows the formation of small cloud mist near the ultrasonic horn (indicated with red arrow) after the transducer was turned on, which later on forms into a large cavitation cloud covering the whole of the sonotrode surface (Fig. 10c). As this cloud collapses in the next frame (t=4.34 ms), a small notch starts to appear at the bottom of the crystal (encircled in blue). Note that since these primary crystals are highly brittle, a crack in any location of the crystal will form depending on the amount of internal flaws that generally act as stress raisers and crack initiation sites. As the cycle of cloud formation and collapse repeats from t=4.40 ms to t=4.68 ms, the crack grows and propagates until it reaches the edge of the crystal (shown in blue rectangular border). Subsequently the crystal fails as the new crack connects to the existing crack (enclosed in a green boundary). From the images, it is apparent that there has been no direct interaction of the cavitation cloud with the studied crystal till t=5.02 ms while the fracture being already initiated. It should be noted that the crystal fragmentation in this case occurs in a very similar fashion as was observed in the case of single bubble interaction. The existing crack propagates and then splits into two secondary crack branches, indicating that the failure occurs through combined (opening and tearing) fracture modes. Unlike the laser-induced bubble, however, the fragmentation here is not instantaneous and occurs in about 7 ms (168 acoustic cycles). It was previously shown that interaction of individual bubbles with intermetallics can be the driving mechanism of fragmentation [21]. It is unlikely that microbubbles of size in the range of few microns (which cannot be resolved by the camera lens) oscillating far away from source, are capable of damaging the crystal and causing catastrophic fracture in such a short span of time. Indeed, microbubbles with radii in the range of 10–15 μm produces pressure pulses close to 0.1 MPa upon transient collapse that is insufficient to induce fast fracture of the crystal [29], [62]. Wang et al. [21] previously reported that large pulsating cavitation bubbles with radii in the range of 50–80 μm that undergo vigorous periodic collapses, damages the nearby crystal after a significant period of 1680 acoustic cycles. So, even if there are non-linearly oscillating bubbles with radii in the range of 10 μm or less attached to the crystal for longer duration, it is unlikely that they would induce severe damage in such a short period of time as they are not likely to collapse [62], [63]. Based on the number of acoustic cycles, it is conceivable that the crystal must have survived almost 100 shock waves (based on the shock front approximation as per Fig. 9) prior to fracture. It appears that, even though the fragmentation of crystal as observed in the section 3.2.1 is almost instantaneous, the Al_3_Zr intermetallic fracture can also be affiliated with fatigue type failure in view of the fact that the crystal survived multiple cavitation cloud collapses before its complete rupture. It is suggested that emitted shock waves from cavitation cloud collapses initially caused the formation of a sub-critical crack that later developed into a critical size crack as observed by Wang et al. [21]. Results show, for the first time, that the governing mechanism is due to the shock waves generated from clouds of bubbles near the sonotrode tip, which may also be complemented by the vigorous oscillation of individual pulsating cavitation bubbles in the vicinity of the crystal.

**Fig. 11:**
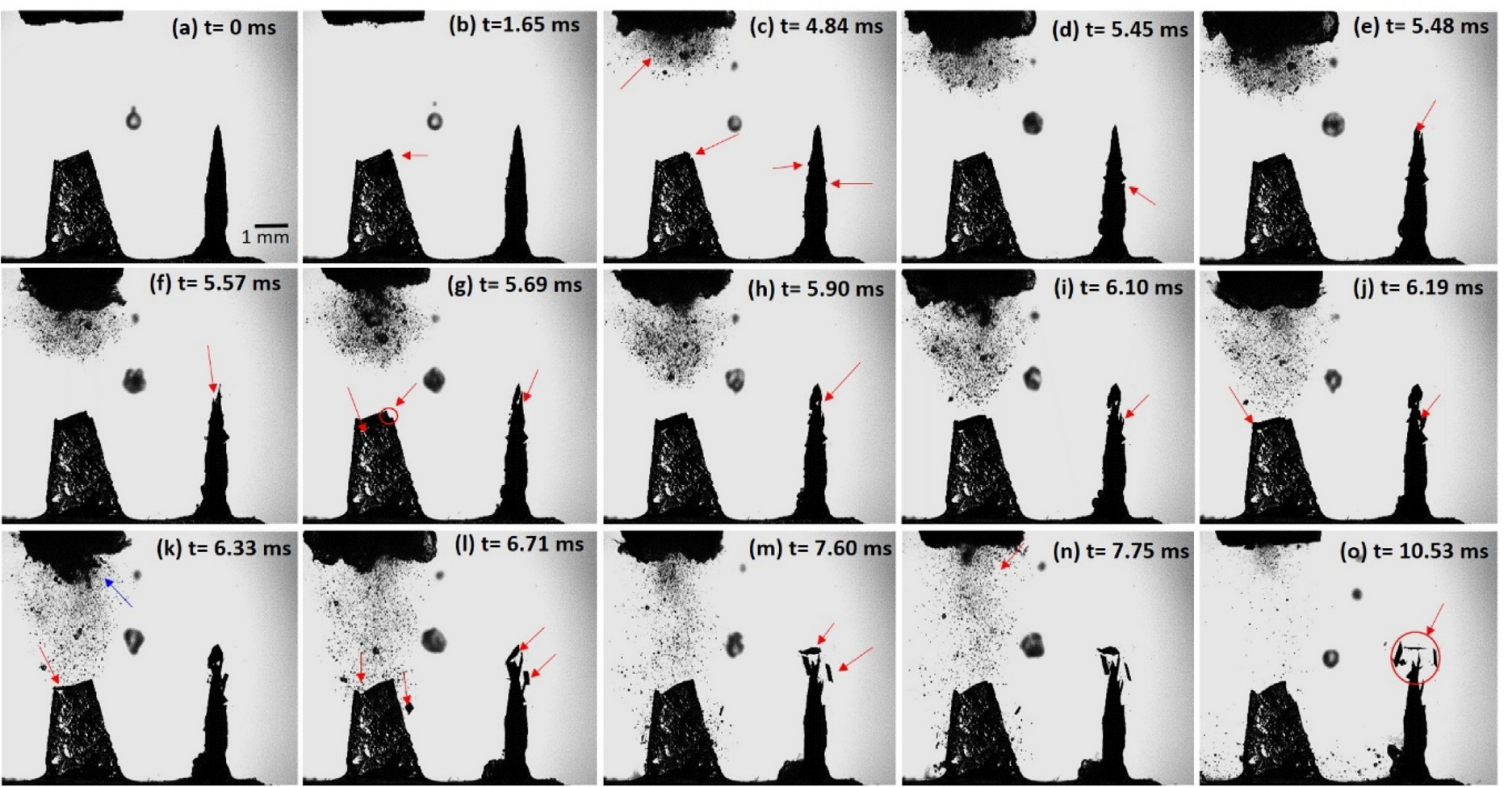
High-speed images of fragmentation of primary Al_3_Zr crystals captured at 100 kfps when subjected to ultrasonic cavitation. The supplementary video can be accessed with the online version of the article as video 4.

In addition to the evaluation of the fracture mechanism of an intermetallic crystal having a pre-existing notch, the fragmentation sequence has also been observed for a perpendicularly oriented crystal placed at roughly 3 mm vertically below and around off-axis 4 mm in the crosswise direction, to account for any bending effects of the fixed crystal from the ultrasound-induced acoustic cavitation pressure field. Again, to avoid any uncertainty caused by microbubbles attached to the crystal’s surface as in the case of a crystal in Fig. 10, sonication was applied only after making sure that there are no pre-existing bubbles touching the intermetallic that can be resolved by the camera lens. Fig. 11(a-o) shows the sequence of sono-fragmentation images of forward-facing and side-facing primary Al_3_Zr crystals. An associated video corresponding to this sequence of images (File name: video 4) is accessible with online version of the article. Only selected frames have been shown for the best representation of the facture mechanism and brevity of the manuscript.

The first image at t=0 ms displays the well-illuminated crystals placed in two perpendicular planes below the 3 mm sonotrode tip. For clarity, the crystal on the left will be termed as CL and crystal on the right as CR from here onwards. At t=1.65 ms, a few tiny bubbles started to appear just beneath the sonotrode and a tiny crack is formed at the top of the CL (indicated by red arrow). After about 3.19 ms, clusters of bubble start to cavitate resulting in the possible release of shock waves causing the crack formed on CL to enlarge and notches on CR to form (Fig. 11c). At t=5.45 ms, a notch on CR has developed upon increased exposure time of ultrasound and can be clearly seen. Then at t=5.9 ms (Fig. 11*h*), the top right corner of CL has completely separated off and as the propagating bubble cloud becomes denser, the crack starts to develop on CL and a visible cavity forms at the top of the CR (see supplementary video 4 for clarity). Note that the position where the crack, notch or cavity develops first in the crystal depends, apparently, on the presence of a pre-existing micro crack and defects at those locations, which cannot be resolved in the images. In Fig. 11(h to k), as the cavitation bubble cloud approaches the crystals, the top portion of the CR exhibits severe flapping, followed by the fragmentation and a top left corner of CL is detached (indicated by the arrow). The flapping part of the CR ultimately shears away as shown in Fig. 11(o). Also, it can be noticed from the sequence of snapshots that a relatively large cavitating bubble in the centre of the images (and within depth of field of 6 mm) has been captured by a standing wave and trapped at the same location changing its vibration (surface/Faraday waves) modes [64] in a stable non-linear motion throughout the sequence without significant variation in its size, indicating the weak effect of the incident pressure waves at that specific position. This confirms evidence that there is almost negligible effect of incident waves and acoustic streaming on the CR located away from the source. Hence, only the powerful surges from shock waves due to the bubble cloud collapse can be responsible for shattering the CR as in Fig. 11o.

The Al_3_Zr crystal (CR) underwent just 186 cycles of ultrasonic vibration before its complete fragmentation representing low cycle fatigue type failure. It is worth mentioning that the fracture of crystals at certain locations occurred before the cavitation cloud even approached the intermetallic surface. Also, there is no evidence of a pulsating bubble clusters on the surface of the crystal that can possibly induce damage as a result of liquid jet ejection. So, the only likelihood is that the fragmentation occurs through shock wave interaction causing the crystal to shatter in pieces like that of glass particularly when it is outside the cavitation zone. These observations confirm that the shock waves emitted from the collapse of cavitating bubble cloud act as a crucial agent responsible for the fragmentation of primary crystals, as evaluated in next section. In addition, repeated emission of shock waves impacting the crystal surface intermittently cause low cycle fatigue within the material, eventually fragmenting the crystal thus making the cyclic shock pressure one of the possible fracture mechanism just before the brittle failure [21].

#### 3.3.2 Analysis of induced shear stresses on the studied crystals

As observed from Fig. 10 and Fig. 11, it is apparent that the acoustically generated large cavitation structure attached to the sonotrode undergoes repetitive dynamic oscillations. It is interesting to note that the oscillating frequency of intermetallic deflection decreases to almost 4.8–5 kHz just before the fragmentation (from t=4.81 ms to t=5.62 ms at steps of 0.2 ms) matching the self-induced subharmonic frequency (1/5th of the driving frequency) of the large cavitation structure attached to the sonotrode tip as shown in Fig. 11*k*. This sub-harmonic oscillation of the crystal is indirectly related with the emission of shock waves every time the attached cavitation cloud collapses [65]. The strange behaviour of this large formed cavitation structure has previously been observed by Znidarcic et al. [66]. It was reported that this developed cavitation structure generates its own oscillating frequency that falls in the subharmonic range of the ultrasonic driving frequency. The strength of this subharmonic signal induced from this large cloud can actually be very high and its acoustic intensity in the power spectrum can even come close to the fundamental pressure wave [67]. Johnston et al. [68] found that these strong subharmonic signals are related to the emissions of periodic shock waves from acoustically driven cavitation cloud collapses.

**Fig. 12:**
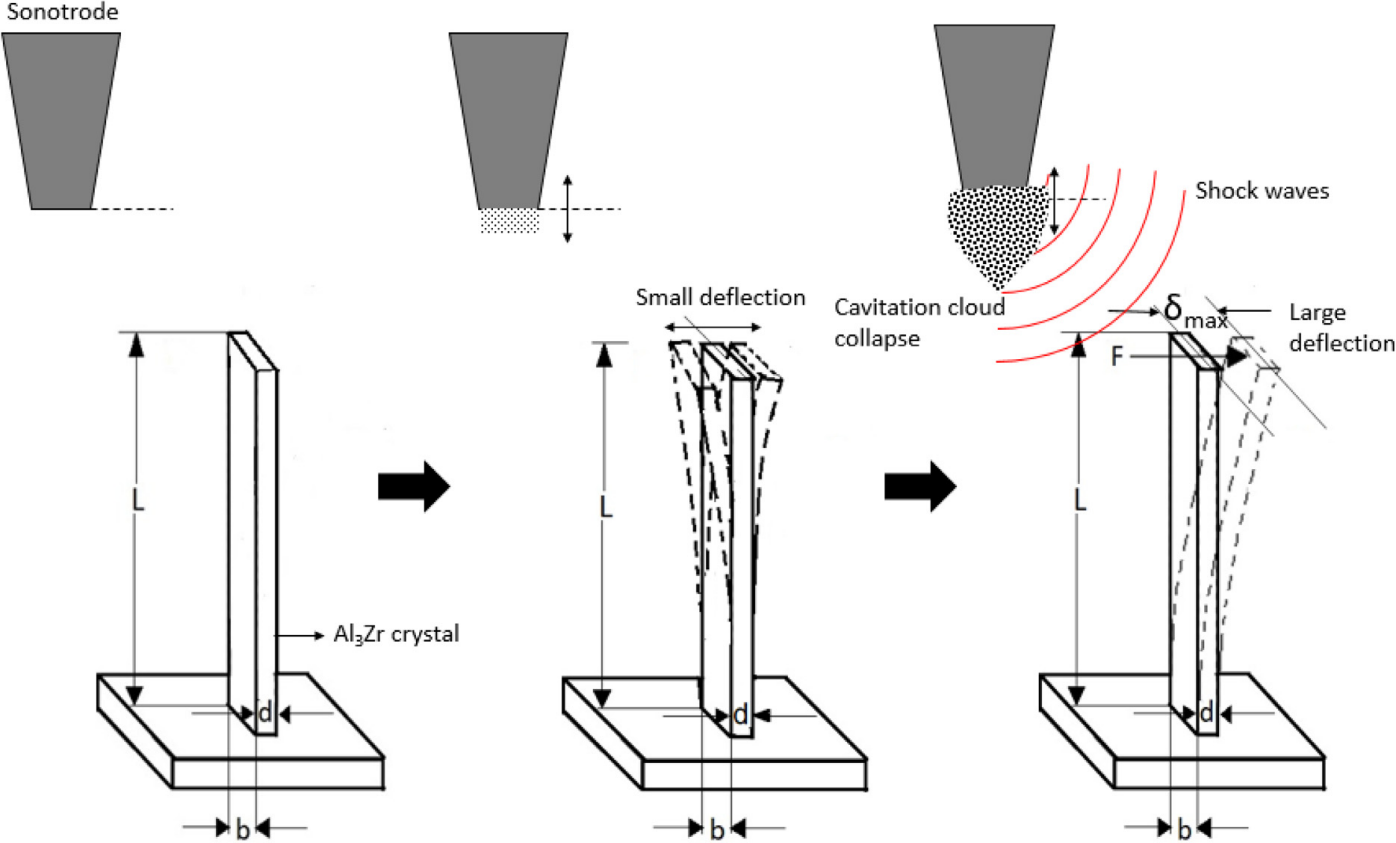
Schematic representation of intermetallic crystal deflecting upon interaction with the incoming shock waves.

The impact of released shock waves on the CR intermetallic crystal has been explained at different time progressions (see supplementary video 4 for better clarity). As soon as the ultrasound is switched on, the CR undergoes continuous deflection of about 24.3 μm at t=1.05 ms oscillating close to the driving frequency (24 kHz) with simultaneous build-up of cavitation structure near the sonotrode. It was observed that at specific time steps where the bubble cloud collectively collapses, the pressure impulse from the generated shock wave causes the maximum deflection of the crystal viz. t=3.62 ms, t=5.07 ms, t=5.28 ms and t=5.62 ms. This peculiar behaviour has been schematically exemplified in Fig. 12 for clarity. The cavitation cloud reaches its maximum size at t=5.48 ms and with subsequent collapses the CR intermetallic shatter into pieces at t=6.71 ms with fragments as small as 50 μm. This phenomenon of potential refinement of the primary intermetallic crystals in ultrasonically treated real melt systems is very important in grain refinement with these fragments acting as grain nucleation points without need of any separate inoculation [8].

This shock wave-induced maximum deflection of CR can be utilised to mathematically estimate the shear stress acting at the tip of the crystal. For calculation, the length (*L*), width (*b*) and thickness (*d*) of CR shown in Fig. 12 are taken as 3.6 mm, 2.5 mm and 0.06 mm, respectively. The maximum shear stress (*τ_max_*) acting on the crystal was evaluated by adopting a simple cantilever beam model using the following equations as described in [69]:

(4) $$\tau_{\max}=\frac{\mathrm{FQ}}{\mathrm{Ib}}$$

(5) $$F=\frac{3\delta_{\max}EI}{L^{3}}$$

(6) $$Q=\frac{bd^{2}}{8}$$

(7) $$I=\frac{bd^{3}}{12}$$

Here *F* is the transverse shear force estimated from the maximum deflection of the crystal (*δ_max_*), *Q* is the first moment of area at the neutral axis, *I* denotes the moment of inertial of the crystal’s cross-section and *E* is elastic modulus of the Al_3_Zr crystal taken from Table 1. Based on the above equations, *τ_max_* was calculated for different deflection values found at specific time intervals and is shown in Table 2. As we can see, the induced shear stress ramps up with the crystal’s deflection reaching up to 0.5 MPa at the point of maximum bending of the tip. A similar approach was also undertaken by Kim et al. [63] where a silicon microcantilever was subjected to oscillating bubbles in a continuous ultrasonic field and its damage potential was assessed through deflection-induced stresses. Their study confirms that deflection of the crystal is not due to acoustic streaming or the developed pressure field, but rather it is driven by the dynamic pressure effects from chaotically oscillating bubble collapses. Interestingly, the deflection produced by the chaotic oscillating bubbles with the pressure in the range of 0.1–0.5 MPa is sufficient to break the Si cantilever, matching very well our findings. Zeng et al. [70] also reported that the cavitation bubble collapse near a rigid solid boundary induces shear stresses in the range of 100 kPa upon liquid jet impact. Even if there is a stable pulsating microbubble attached to the crystal’s surface as observed by Kim et al. [63], based on the observed free end deflection (*δ*), the induced bubble pressure exerted over the cantilever beam length (*L*) given by $P=2\delta Ed^{3}/3L^{4}$, is about 0.4 kPa. Thus, the imparted pressure by the attached microbubble is almost three orders of magnitude lower than the induced shear stress by the shock waves and liquid jet emitted from the chaotically oscillating bubble cloud just beneath the sonotrode surface. This estimate clearly indicates that the pressure fluctuation due to microbubble undergoing oscillations at the crystal surface is unlikely to induce any rapid damage.

**Table 2:** Calculated maximum shear stress based on the crystal’s free end deflection measured at different time intervals.

**t (ms)**	**δ_max_ (μm)**	**τ_max_ (kPa)**
1.05	24.3	141.1
3.62	48.7	282.2
5.07	73.1	423.4
5.28	97.5	564.5

This experiment can also be related to the observations of fragmentation of elongated dendrites in real Al-Cu melts observed in [12]. However, in contrast to study [12] where acoustic flow were suggested to be responsible for the fragmentation of fine and micro-size (5–60 μm) dendrites, we suggest that the stress induced by the acoustic streaming is not sufficient to break-up the crystal via deflection. We have recently showed that acoustic streaming velocities in the range of 0.1–0.3 m/s (typical range from a sonotrode-induced flow in water and in liquid aluminium) generate very small flow pressure fields certainly not adequate to deflect and break the crystal [71]. It is worth noting that there are two factors that differ our study with that of Wang et al. [12], i.e. in their study the crystals were long and thin (different length/thickness ratio from ours) and the fragmentation in the melt could be assisted by local remelting (which does not take place in our study).

### 3.4 Acoustic pressure measurements of the emitted shock waves and application to fragmentation

**Fig. 13:**
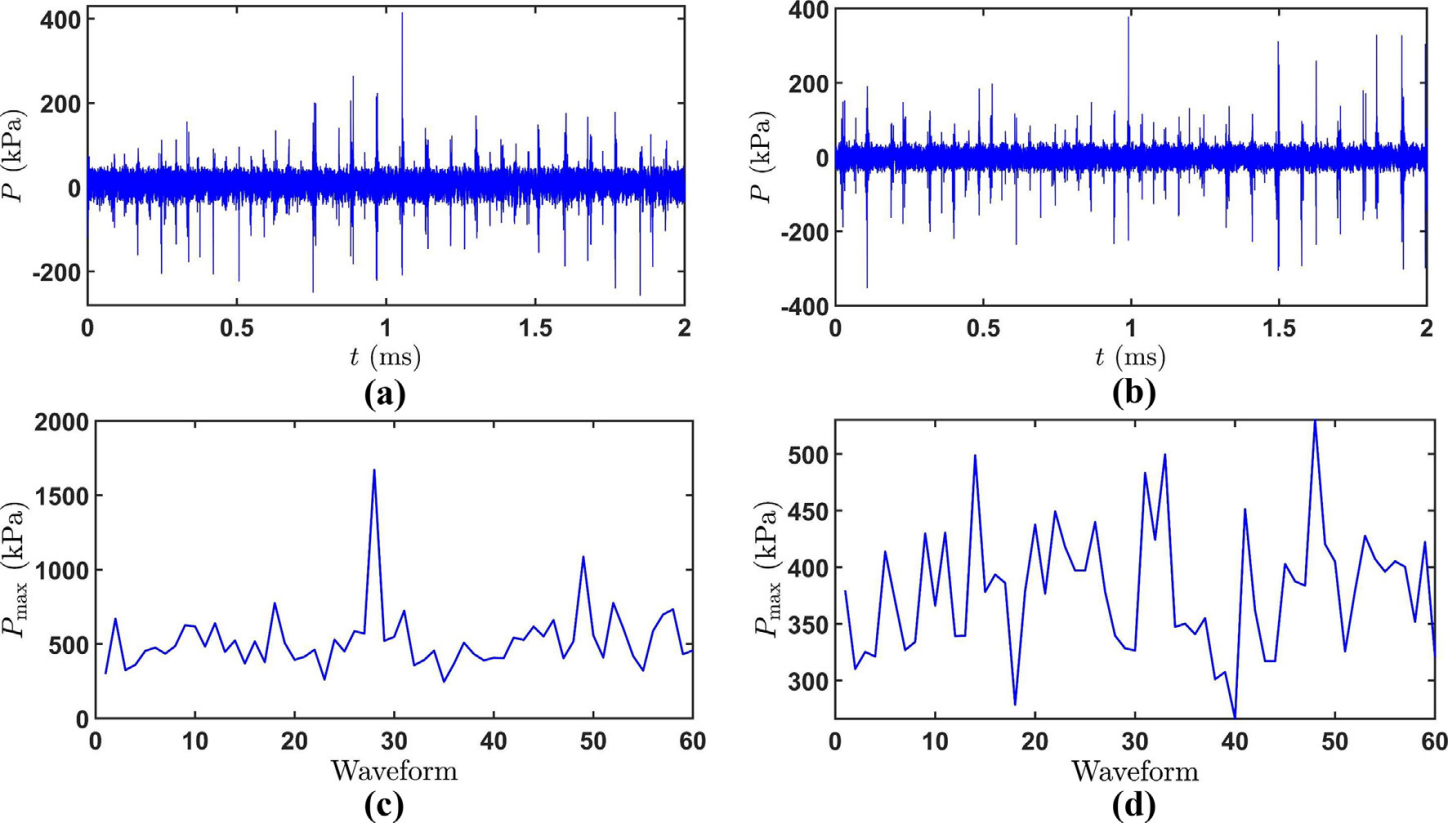
Time evolution of pressure at locations (a) under and (b) at the side of sonotrode, maximum pressure (c) and (d) recorded for 60 acoustic waveforms points corresponding to (a) and (b) respectively, measured using fibre optic hydrophone.

The intermetallic fragmentation has also been complemented by measuring the pressure magnitude of the travelling shock waves in the vicinity of the crystals. Fig. 13 shows the temporal and peak pressure distribution of the propagating shock fronts, captured using a highly sensitive and calibrated fibre optic hydrophone (section 2.5). Quantitative analysis of the pressure distribution in the time domain (Fig. 13a & 13b) indicates the peak-positive pressure amplitude from the corresponding shock waves. Note, however, that the negative phase has been preserved. The apparent negative phases are most likely associated with the lack of phase calibration (experimental bubble collapse shock wave profiles are magnitude-only deconvoluted) of the fibre optic hydrophone. Although, phase changes may result in alterations of the shape of time domain pressure signal (Fig. 13a & 13b), they do not affect the integrated energy contained within it and consequently the pressure magnitude (Fig. 13c & 13d) [37]. This is evident by the work of Johansen et al. [25] where it was experimentally shown that in a cavitating environment, the pressure magnitude is only 3% higher in a deconvoluted phase and magnitude profile than those of magnitude-only deconvoluted profiles. So, for this study, where we are interested on the actual peak pressure values in relation to the fragmentation mechanism of intermetallics, the phase calibration of hydrophone is not essential and is out of scope. Additionally, negative peaks may also be related to the reflection of shock fronts with the bubbly clouds and the myriads of various size bubbles in the bulk liquid as suggested by [72]. The peak maximum pressure (*P_max_*) averaged across 60 generated waveforms of 2 ms period each for crystals CL (under) and CR (side) in Fig. 13c and Fig. 13d was measured to be 523 kPa and 380 kPa respectively. However, pressure surges of up to 1.6 MPa were also observed and these are in a very good agreement with the calculated shear stresses induced by the deflection of the fixed crystal (Table 2). Therefore, it can be inferred that the pressure exerted by the repetitive shock waves emitted from the sub-harmonic frequency pulsation of the large cavitation cloud generating impulsive loads with peak pressures in the range of 0.4–0.5 MPa can induce sufficient shear stress to break the studied crystals. It should be noted that shock wave propagation remains virtually undisturbed in the liquid medium at the sides of the ultrasonic horn (Fig. 9 video 2). Whereas, under the sonotrode, the cavitation zone is exceedingly populated with numerous bubbles collapsing and self-interacting with each other. So even if some of the propagating shock fronts get absorbed or lose their energy, on a cumulative basis they are still sufficient in numbers to fragment the weak regions within the crystal present underneath. The pressure of the shock front in the ultrasonic environment is likely to be affected due to the development of cavitation shielding [36], the decrease in the speed of sound and the density of the surrounding medium owing to the presence of a large bubble cloud (half sphere) attached to the ultrasonic horn and other individual cluster or bubbles in the bulk liquid [73]. The observation of this acoustically induced large cavitation structure as it undergoes violent collapses, has been referred to as the phenomenon of acoustic supercavitation elsewhere [66]. It has also been found that the shock pressure induced from the large bubble cloud of radius ~ 1.3 mm is similar to our observation (Fig. 10c and Fig. 11*k*) and is three times higher than the pressure measured in the case of single bubble collapse measured at a distance of 10 mm from the ultrasound focus [53]. This clearly indicates that the location of crystals also plays a significant role in producing effective and instantaneous fragmentation. However, since the crystal is placed further away from the acoustic horn, the registered pressure is much lower. Pressure generated from the large cavitation structure attached to a 20 kHz horn tip (Ø=3 mm) was found to reach up to 250 kPa during its collapse [66] at a distance of 7 mm, which seems to agree reasonably well with our acoustic pressure measurements.

As discussed in section 3.2.2, the stress required for intermetallic fracture lies in the range of 20–40 MPa depending upon the existing crack length and associated defects. The shock wave emission from a single bubble collapse of ~ 1.5 mm radius also produces pressure of a similar order, causing instantaneous fragmentation. However, the fragmentation of the crystal with (Fig. 10) and without (Fig. 11) a pre-existing crack under the presence of ultrasound is not instantaneous and occurs between 150 and 200 acoustic cycles representing fatigue type failure. Results are in very good agreement with the work of Wang et al. [21] where, even if fragmentation occurred in an order of time higher (1680 cycles) as the location of intermetallic crystal was further away (~4 mm) from 1 mm sonotrode tip (thus less input energy and shock wave emission), the fatigue mechanism due to the cyclic acoustic pressure was also suggested. According to Wang et al. [21], the probable reason for this prolonged breaking time of crystal corresponds to the emitted pressure waves from the individual pulsating bubbles with radii in the range of 50–80 μm which decay significantly with increasing distance. Specifically, a recent study by Pishchalnikov et al. [23] reported that a bubble of a similar size as observed in [21] generates pressure as high as 50 MPa at a distance of about 30 μm from the collapsing spot. This essentially indicates that for instantaneous breakage to happen (as in the case of the laser-induced bubble experiments), the bubble collapse should occur immediately next to the crystal. Moreover and according to Ref. [23], pressure surges in the range of 1.6 MPa as measured with the fibre-optic hydrophone in our study can be achieved at a distance of 100–150 μm from the point of the bubble collapse. So, if an individual bubble of a few tens of microns size collapses beyond this distance, the crystal is not likely to be damaged in a short period of time. However, in the case of a sonotrode where a large cloud of bubbles instantly collapses (Fig. 10c-10d), a much stronger pressure wave or an instrumental collection of pressure surges impact the specimen. This action is further intensified by the fast acceleration of the sonotrode’s mechanical action [66]. Therefore, the incoming multiple shock wave pressures from large cavity collapses near the sonotrode tip are indeed much stronger and carry substantially more energy than the individual stable or transient pulsation/collapse, causing crystal fragmentation at even large distances in just a few tenths/hundreds of acoustic cycles.

Based on the crack length (Fig. 10) of 1.8 mm, the required stress for the complete fracture is estimated to be approximately 13 MPa as per Eq. (3). Although the stress needed is much higher than the produced acoustic pressure, the fragmentation still happens due to constant interference with propagating shock waves. In statistical terms, and based on Fig. 9 (video 2) observations, the crystal CL and CR in Fig. 11 experiences about 100 repetitive and low amplitude shock wave shots, while still strong pressure surges in the range of 0.1 to 0.4 MPa impact the intermetallic surface causing the crystal to fail within 10 ms. Besides, the sono-fragmentation in all the studied crystals occurred in between 5 and 10 ms. This period of time relates to a few smaller pressure peaks, about 100–200 (~40 peaks every 2 ms according to Fig. 13a & 13b) in the range of 50–200 kPa and at least 5 maximum pressure surges from the collapsing bubbles in the range of 0.5 to 1.5 MPa according to Fig. 13c & 13d. Therefore, it is reasonable to say that stresses pile-up on the intermetallic surface under continuous interaction with the ultrasound-induced low-pressure shock waves, generating fatigue in the material that eventually lead to catastrophic failures.

The fragmentation mechanism in a real melt system also bears similarity to our observations. It was reported in [11] that a single bubble implodes in just one acoustic cycle near the ultrasonic source and the breaking up of the solid phase is accelerated by such collapsing bubbles and the high speed acoustic flows in the range of 1–2 m/s. However, this effect may weaken as one moves further away from the ultrasonic source and the cavitation bubbles may survive tens and thousands of acoustic cycles owing to the exponential decrease in the acoustic cavitation intensity with distance [32] and attenuation and sound absorption in the cavitation region [13]. In regions, away from the sonotrode, the fragmentation mechanism of solidifying phases relates to the cyclic fatigue effect induced by the pulsating bubbles and momentum of acoustic flow (0.05–0.3 m/s) [71]. Wang et al. [11] predicted that pressure amplitude induced by oscillating bubbles in a Bi-8% Zn alloy melt was mostly between 20 and 30 MPa that was capable to fragment the free floating primary Zn phase in about 3000–5000 acoustic cycles. It has been found that certain intermetallics tend to show a more plastic behaviour (increased yielding and flow stresses) with increasing temperatures thus creating resistance to breakage [74], [75]. However, in our case the pressure and time required for fragmentation seems to be much lower considering that the studied intermetallic was different and sonication was performed in water at room temperature. We also anticipate that fragmentation will be much faster in liquid Al than actually predicted, due to higher and more prominent dynamics of the cavitation bubbles [19], [36] as well as the fact that the crystal will interact with the cavitation zone more frequently as it is likely to be freely floating. Local remelting at necking and initial cracks may further assist fragmentation of intermetallics in the melt.

Following the elucidation of the governing fracture mechanism of primary aluminium crystals, the authors firmly believe that the high-speed in-situ real time sono-fragmentation imaging, detailed in this article, provides substantial understanding of the fragmentation induced intermetallic crystal refinement upon UST of real metallic melts. All the experiments discussed in this paper were performed by fixing the extracted primary crystals onto a solid substrate. However, this may not be the case in real Al melts where primary crystals also exist in a free-floating condition. It is believed that the mechanism observed for the fixed primary particles is still valid considering the high frequency repetitions of cyclic pressures and the short impact application duration. The catastrophic fracture occurs in very short period of time and within the very first millisecond depending on the position of the sample (the closer to the sonotrode the faster the rapture should be) as well as the deflection that occurs as intermetallics grows as previously discussed in [11].

This study also highlights that shock waves emitted from the active cavitation zone (just below the sonotrode surface) travel up to considerable distances carrying sufficient potential energy to damage intermetallic crystals upon impact; this is unlike other studies [21], [23], [29], [70] which demonstrate that cavitation bubbles should be in very close proximity to the solid boundaries in order to produce severe damage. Thus, the limits of the active cavitation zone can be potentially quantified and optimised promoting effective cavitation treatment. Apart from metallurgical applications, the results of this study are of significance for applications in relevant scientific fields such as surface cleaning [76], bacterial cleaning and water decontamination [77], exfoliation of 2D nanomaterials [78] as well as tumour treatment [79] and medical lithotripsy [23] dealing with cavitation bubbles’ interaction with solid surfaces.

## 4 Conclusions

In this study, interactions of single and multiple cavitation bubbles against fixed individual intermetallic Al_3_Zr crystals with quantifiable mechanical properties were investigated. After analysing the real time recorded high-speed images of cavitation bubbles interaction with the studied samples, the governing mechanism of Al_3_Zr fragmentation was elucidated. The following major findings of this research are:

1. Controlled interaction of laser-induced cavitation bubbles with the intermetallic crystals revealed that the governing fragmentation mechanism is the exerted shock wave pressure upon bubble collapse.
2. In a continuous sonicated environment, crystals should not necessarily be in the vicinity of the collapsing bubbles for fragmentation to occur. Fragmentation occurs in a range of distances from the acoustic source and involves two-steps: (i) the propagating shock wave fronts continuously interfere with the nearby intermetallic crystals causing a low cycle fatigue (around 100–200 shocks arriving the sample surface in less than 10 ms) until the existing crack reaches its critical length; (ii) fragmentation occurs immediately afterwards, via brittle fracture.
3. In the case of a laser-induced cavitation bubble, the pressure amplitude required for the instantaneous fragmentation of the intermetallic samples is in the range of 20–40 MPa based on critical crack length measurements. In contrast, in the case of ultrasonic cavitation, the fragmentation occurs in between 150 and 200 acoustic cycles, indicating a low fatigue cycle mode with surges of acoustic pressures from corresponding shock waves in the range of 0.4–0.5 MPa.
4. For a crystal with an existing crack length, the crack propagation occurs both in and out of the shear plane indicating that the crack separation happens through combined fracture modes.
5. The repetitive sub-harmonic collapse of a large cavitation structure attached to the ultrasonic horn is responsible for repeated bending and brittle fracture of the fixed intermetallic crystals, relevant to that of the growing dendrites in the solidification front in a real melt, with corresponding shear stresses in the range of 0.1–0.5 MPa.

Future work will focus on the determination of temporal and spatial relationships of the ultrasound-induced fragmentation of primary crystals that can act as a vital base line to obtain efficient grain refinement in a real melt system. The effect of the extension of cavitation zone as well as the critical residence time on the microstructural damaging mechanism and fragment size distribution of free floating intermetallics will be explored.

On the other hand, the use of water as a transparent analogue to liquid Al, still does not exactly replicate the real conditions in a liquid melt. However, based on previous work from our group where the cavitation dynamics in water and liquid aluminium were studied, it can be confidently deduced that due to the much higher aggressiveness of cavitating environment in liquid aluminium, perhaps crystals will fail even faster. This still remains a subject for further research and we are planning to measure the mechanical properties of intermetallics under high temperature conditions using the same nano-indenter described in section 2.2 and perform in-situ fragmentation experiments of free floating intermetallics in melt conditions using a synchrotron facility.

## CRediT authorship contribution statement

**Abhinav Priyadarshi:** Conceptualization, Methodology, Software, Validation, Formal analysis, Investigation, Resources, Writing - original draft, Writing - review & editing. **Mohammad Khavari:** Software, Formal analysis, Investigation. **Tungky Subroto:** Methodology, Resources. **Marcello Conte:** Investigation. **Paul Prentice:** Methodology, Resources, Supervision. **Koulis Pericleous:** Writing - review & editing, Supervision, Funding acquisition. **Dmitry Eskin:** Writing - review & editing, Supervision, Funding acquisition. **John Durodola:** Supervision. **Iakovos Tzanakis:** Conceptualization, Methodology, Resources, Writing - review & editing, Supervision, Funding acquisition.

## Declaration of Competing Interest

The authors declare that they have no known competing financial interests or personal relationships that could have appeared to influence the work reported in this paper.

## References

[1] Eskin D.G., Mi J. (2018). Solidification Processing of Metallic Alloys Under External Fields.

[2] Eskin D.G., Tzanakis I., Wang F., Lebon G.S.B., Subroto T., Pericleous K., Mi J. (2019). Fundamental studies of ultrasonic melt processing. Ultrason. Sonochem..

[3] Eskin G.I., Eskin D.G. (2015). Ultrasonic Treatment of Light Alloy Melts.

[4] Campbell J. (1981). Effects of vibration during solidification. Int. Met. Rev..

[5] W. Kurz, D.J. Fisher, Fundamentals of Solidification, Trans Tech Publication, Zurich, Switzerland, 1998. https://www.scribd.com/document/353281924/81869805-Fundamentals-of-Solidification-W-Kurz-D-J-Fisher-4th-pdf (accessed August 2, 2019).

[6] Zhang L., Eskin D.G., Katgerman L. (2011). Influence of ultrasonic melt treatment on the formation of primary intermetallics and related grain refinement in aluminum alloys. J. Mater. Sci..

[7] Eskin G.I., Eskin D.G. (2003). Production of natural and synthesized aluminum-based composite materials with the aid of ultrasonic (cavitation) treatment of the melt. Ultrason. Sonochem..

[8] Atamanenko T.V., Eskin D.G., Zhang L., Katgerman L. (2010). Criteria of grain refinement induced by ultrasonic melt treatment of aluminum alloys containing Zr and Ti. Metall. Mater. Trans. A..

[9] Eskin G.I., Eskin D.G. (2004). Some control mechanisms of spatial solidification in light alloys. Zeitschrift Für Met..

[10] Eskin G.I. (2001). Broad prospects for commercial application of the ultrasonic (cavitation) melt treatment of light alloys. Ultrason. Sonochem..

[11] Wang B., Tan D., Lee T.L., Khong J.C., Wang F., Eskin D., Connolley T., Fezzaa K., Mi J. (2018). Ultrafast synchrotron X-ray imaging studies of microstructure fragmentation in solidification under ultrasound. Acta Mater..

[12] Wang F., Eskin D., Mi J., Wang C., Koe B., King A., Reinhard C., Connolley T. (2017). A synchrotron X-radiography study of the fragmentation and refinement of primary intermetallic particles in an Al-35 Cu alloy induced by ultrasonic melt processing. Acta Mater..

[13] Xu W.W., Tzanakis I., Srirangam P., Mirihanage W.U., Eskin D.G., Bodey A.J., Lee P.D. (2016). Synchrotron quantification of ultrasound cavitation and bubble dynamics in Al-10Cu melts. Ultrason. Sonochem..

[14] Xu W.W., Tzanakis I., Srirangam P., Terzi S., Mirihanage W.U., Eskin D.G., Mathiesen R.H., Horsfield A.P., Lee P.D. (2015). In situ synchrotron radiography of ultrasound cavitation in a molten Al-10Cu alloy. in TMS2015 Supplemental Procedings.

[15] Swallowe G.M., Field J.E., Rees C.S., Duckworth A. (1989). A photographic study of the effect of ultrasound on solidification. Acta Metall..

[16] Chow R., Blindt R., Kamp A., Grocutt P., Chivers R. (2004). The microscopic visualisation of the sonocrystallisation of ice using a novel ultrasonic cold stage. Ultrason. Sonochem..

[17] Shu D., Sun B., Mi J., Grant P.S. (2012). A high-speed imaging and modeling study of dendrite fragmentation caused by ultrasonic cavitation. Metall. Mater. Trans. A..

[18] Wagterveld R.M., Boels L., Mayer M.J., Witkamp G.J. (2011). Visualization of acoustic cavitation effects on suspended calcite crystals. Ultrason. Sonochem..

[19] Tzanakis I., Lebon G.S.B., Eskin D.G., Pericleous K.A. (2016). Characterisation of the ultrasonic acoustic spectrum and pressure field in aluminium melt with an advanced cavitometer. J. Mater. Process. Technol..

[20] Tzanakis I., Lebon G.S.B., Eskin D.G., Pericleous K.A. (2017). Characterizing the cavitation development and acoustic spectrum in various liquids. Ultrason. Sonochem..

[21] Wang F., Tzanakis I., Eskin D., Mi J., Connolley T. (2017). In situ observation of ultrasonic cavitation-induced fragmentation of the primary crystals formed in Al alloys. Ultrason. Sonochem..

[22] Priyadarshi A., Subroto T., Conte M., Pericelous K., Eskin D., Prentice P., Tzanakis I., Tomsett A (2020). Nanoindentation and cavitation-induced fragmentation study of primary Al3Zr intermetallics formed in Al alloys. Light Metals 2020.

[23] Pishchalnikov Y.A., Behnke-Parks W.M., Schmidmayer K., Maeda K., Colonius T., Kenny T.W., Laser D.J. (2019). High-speed video microscopy and numerical modeling of bubble dynamics near a surface of urinary stone. J. Acoust. Soc. Am..

[24] Vogel A., Busch S., Parlitz U. (1996). Shock wave emission and cavitation bubble generation by picosecond and nanosecond optical breakdown in water. J. Acoust. Soc. Am..

[25] Johansen K., Song J.H., Johnston K., Prentice P. (2017). Deconvolution of acoustically detected bubble-collapse shock waves. Ultrasonics..

[26] Supponen O., Obreschkow D., Kobel P., Tinguely M., Dorsaz N., Farhat M. (2017). Shock waves from non-spherical cavitation bubbles. Phys. Rev. Fluids..

[27] Brujan E.A. (2019). Shock wave emission and cavitation bubble dynamics by femtosecond optical breakdown in polymer solutions. Ultrason. Sonochem..

[28] Akhatov I., Lindau O., Topolnikov A., Mettin R., Vakhitova N., Lauterborn W. (2001). Collapse and rebound of a laser-induced cavitation bubble. Phys. Fluids..

[29] Ohl S.W., Klaseboer E., Khoo B.C. (2015). Bubbles with shock waves and ultrasound: a review. Interface Focus..

[30] Holzfuss J. (2010). Acoustic energy radiated by nonlinear spherical oscillations of strongly driven bubbles. Proc. R. Soc. A Math. Phys. Eng. Sci..

[31] Tzanakis I., Eskin D.G., Georgoulas A., Fytanidis D.K. (2014). Incubation pit analysis and calculation of the hydrodynamic impact pressure from the implosion of an acoustic cavitation bubble. Ultrason. Sonochem..

[32] Lebon G.S.B., Tzanakis I., Djambazov G., Pericleous K., Eskin D.G. (2017). Numerical modelling of ultrasonic waves in a bubbly Newtonian liquid using a high-order acoustic cavitation model. Ultrason. Sonochem..

[33] Lebon G.S.B., Salloum-Abou-Jaoude G., Eskin D., Tzanakis I., Pericleous K., Jarry P. (2019). Numerical modelling of acoustic streaming during the ultrasonic melt treatment of direct-chill (DC) casting. Ultrason. Sonochem..

[34] Conte M., Mohanty G., Schwiedrzik J.J., Wheeler J.M., Bellaton B., Michler J., Randall N.X. (2019). Novel high temperature vacuum nanoindentation system with active surface referencing and non-contact heating for measurements up to 800 °C. Rev. Sci. Instrum..

[35] Hielscher, Instruction manual UP200S/ UP400s, 2007. http://www.bendarygroup.com/images/instruction_manual_up200_400s_2007_ultrasonics.pdf.

[36] Lebon G.S.B., Tzanakis I., Pericleous K., Eskin D. (2018). Experimental and numerical investigation of acoustic pressures in different liquids. Ultrason. Sonochem..

[37] Hurrell A.M., Rajagopal S. (2017). The practicalities of obtaining and using hydrophone calibration data to derive pressure waveforms. IEEE Trans. Ultrason. Ferroelectr. Freq. Control..

[38] Wang J., Shang S.L., Wang Y., Mei Z.G., Liang Y.F., Du Y., Liu Z.-K. (2011). First-principles calculations of binary Al compounds: Enthalpies of formation and elastic properties. Calphad..

[39] Oliver W.C., Pharr G.M. (1992). An improved technique for determining hardness and elastic modulus using load and displacement sensing indentation experiments. J. Mater. Res..

[40] Oliver W.C., Pharr G.M. (2004). Measurement of hardness and elastic modulus by instrumented indentation: Advances in understanding and refinements to methodology. J. Mater. Res..

[41] Pal J.W., Jen F.L. (2007). A new method developed for brittle and ductile materials to evaluate mechanical properties of a lump specimen in the use of indentation test. J. Eng. Mater. Technol. Trans. ASME..

[42] Nakamura M., Kimura K. (1991). Elastic constants of TiAl3 and ZrAl3 single crystals. J. Mater. Sci..

[43] Li C., Cheng N., Chen Z., Xie Z., Hui L. (2018). Intermetallic growth and interfacial properties of the grain refiners in Al alloys. Materials (Basel)..

[44] Fine .E., Ghosh G., Isheim D., Vaynman S., Keith Knipling K., Liu J.Z. (2006). Final report for Department of Energy Grant No. DE-FG02-02ER45997, “Alloy Design of Nanoscale Precipitation Strengthened Alloys: Design of a Heat Treatable Aluminum Alloy Useful to 400C.

[45] Fleischer R.L., Zabala R.J. (1990). Mechanical properties of diverse binary high-temperature intermetallic compounds. Metall. Trans. A..

[46] De Fontaine D. (1994). Cluster approach to order-disorder transformations in alloys. Solid State Phys..

[47] Lawn B., Wilshaw R. (1975). Indentation fracture: principles and applications. J. Mater. Sci..

[48] Evans A.G., Charles E.A. (1976). Fracture toughness determinations by indentation. J. Am. Ceram. Soc..

[49] Hu H., Zhao M., Wu X., Jia Z., Wang R., Li W., Liu Q. (2016). The structural stability, mechanical properties and stacking fault energy of Al3Zr precipitates in Al-Cu-Zr alloys: HRTEM observations and first-principles calculations. J. Alloys Compd..

[50] Oriah Bidin N. (1995). Shock wave emission during cavitation bubble collapse in free liquid. Pertanika J. Sci. Technol..

[51] Sinibaldi G., Occhicone A., Pereira F.A., Caprini D., Marino L., Michelotti F., Casciola C.M. (2019). Laser induced cavitation: Plasma generation and breakdown shockwave. Phys. Fluids..

[52] Lauterborn W., Vogel A. (2013). Shock wave emission by laser generated bubbles. Bubble Dyn. Shock Waves.

[53] Brujan E.A., Ikeda T., Matsumoto Y. (2012). Shock wave emission from a cloud of bubbles. Soft Matter..

[54] Janssen M., Zuidema J., Wanhill R. (2004). Fracture Mechanics.

[55] E.J. Hearn, Fatigue, Creep and Fracture, 3rd ed., Butterworth-Heinemann, 1997. https://doi.org/10.1016/B978-075063266-9/50012-3.

[56] Strube H.W. (1971). Numerical investigations on the stability of bubbles oscillating non-spherically. Acta Acust. United with Acust..

[57] Tzanakis I., Hodnett M., Lebon G.S.B., Dezhkunov N., Eskin D.G. (2016). Calibration and performance assessment of an innovative high-temperature cavitometer. Sensors Actuators, A Phys..

[58] Birkin P.R., Offin D.G., Vian C.J.B., Leighton T.G. (2011). Multiple observations of cavitation cluster dynamics close to an ultrasonic horn tip. J. Acoust. Soc. Am..

[59] Jordens J., Appermont T., Gielen B., Van Gerven T., Braeken L. (2016). Sonofragmentation: Effect of ultrasound frequency and power on particle breakage. Cryst. Growth Des..

[60] Zeiger B.W., Suslick K.S. (2011). Sonofragmentation of molecular crystals. J. Am. Chem. Soc..

[61] Kim H.N., Suslick K.S. (2017). Sonofragmentation of ionic crystals. Chem. - A Eur. J..

[62] Ohl C.D., Ikink R. (2003). Shock-wave-induced jetting of micron-size bubbles. Phys. Rev. Lett..

[63] Kim T.H., Kim H.Y. (2014). Disruptive bubble behaviour leading to microstructure damage in an ultrasonic field. J. Fluid Mech..

[64] Versluis M., Goertz D.E., Palanchon P., Heitman I.L., Van Der Meer S.M., Dollet B., De Jong N., Lohse D. (2010). Microbubble shape oscillations excited through ultrasonic parametric driving. Phys. Rev. E - Stat. Nonlinear, Soft Matter Phys..

[65] L. Yusuf, M.D. Symes, P. Prentice, Characterising the cavitation activity generated by an ultrasonic horn at varying tip-vibration amplitudes, Ultrason. Sonochem. (2020) Under review. 10.1016/j.ultsonch.2020.105273 PMC7786551 32795929

[66] Žnidarčič A., Mettin R., Cairós C., Dular M. (2014). Attached cavitation at a small diameter ultrasonic horn tip. Phys. Fluids..

[67] Viet Anh T. (2009). A study of impulsive pressure distribution of cavitation generated by a high-frequency vibrational probe. J. Sci. Technol. (Technical Univ. Pub. Hanoi, Vietnam).

[68] Johnston K., Tapia-Siles C., Gerold B., Postema M., Cochran S., Cuschieri A., Prentice P. (2014). Periodic shock-emission from acoustically driven cavitation clouds: A source of the subharmonic signal. Ultrasonics..

[69] Gere J.M., Goodno B.J. (2013). Mechanics of Materials.

[70] Zeng Q., Gonzalez Avila S.R., Dijkink R., Koukouvinis P., Gavaises M., Ohl C.D. (2018). Wall shear stress from jetting cavitation bubbles. J. Fluid Mech..

[71] Lebon G.S.B., Tzanakis I., Pericleous K., Eskin D., Grant P.S. (2019). Ultrasonic liquid metal processing: The essential role of cavitation bubbles in controlling acoustic streaming. Ultrason. Sonochem..

[72] Maxwell A.D., Cain C.A., Hall T.L., Fowlkes J.B., Xu Z. (2013). Probability of cavitation for single ultrasound pulses applied to tissues and tissue-mimicking materials. Ultrasound Med. Biol..

[73] Yasui K., Iida Y., Tuziuti T., Kozuka T., Towata A. (2008). Strongly interacting bubbles under an ultrasonic horn. Phys. Rev. E - Stat. Nonlinear, Soft Matter Phys..

[74] Koch C.C., Liu C.T., Stoloff N.S. (1985). High-Temperature Ordered Intermetallic Alloys.

[75] I. Baker, D.J. Gaydosh, High-temperature ordered intermetallic alloys II, Materials Research Society, Pittsburgh, PA, 1987. http://inis.iaea.org/search/search.aspx?orig_q=RN:25035800.

[76] Mason T.J. (2016). Ultrasonic cleaning: An historical perspective. Ultrason. Sonochem..

[77] R.A. Torres Palma, E.A. Serna Galvis, Sonolysis, in: Adv. Oxid. Process. Wastewater Treat. Emerg. Green Chem. Technol., Elsevier Inc., 2018: pp. 177-213. https://doi.org/10.1016/B978-0-12-810499-6.00007-3.

[78] Tao H., Zhang Y., Gao Y., Sun Z., Yan C., Texter J. (2017). Scalable exfoliation and dispersion of two-dimensional materials-an update. Phys. Chem. Chem. Phys..

[79] Tu J., Zhang H., Yu J., Liufu C., Chen Z. (2018). Ultrasound-mediated microbubble destruction: A new method in cancer immunotherapy. Onco. Targets. Ther..

